# Combining Brigatinib with mTOR Inhibition to Effectively Treat NF2-SWN–Associated and Sporadic *NF2*-Deficient Meningiomas

**DOI:** 10.1158/2767-9764.CRC-25-0563

**Published:** 2026-01-27

**Authors:** Long-Sheng Chang, Janet L. Oblinger, Lai Man Natalie Wu, Cecelia Miller, Sarah S. Burns

**Affiliations:** 1 Center for Childhood Cancer, Abigail Wexner Research Institute at Nationwide Children’s Hospital, Columbus, Ohio.; 2 Department of Pediatrics, The Ohio State University College of Medicine, Columbus, Ohio.; 3 Department of Otolaryngology-Head & Neck Surgery, The Ohio State University College of Medicine, Columbus, Ohio.; 4 Department of Pathology, The Ohio State University College of Medicine, Columbus, Ohio.; 5 Division of Experimental Hematology and Cancer Biology, Brain Tumor Center, Cincinnati Children’s Hospital Medical Center, Cincinnati, Ohio.

## Abstract

We previously generated an orthotopic, *NF2*-deficient meningioma model using the luciferase-expressing Ben-Men-1 cell line established from a sporadic tumor and identified the multikinase inhibitor brigatinib and the mTOR kinase inhibitor INK128 to potently impede tumor growth. In this study, we describe generation of the telomerase-immortalized AG-NF2-Men cell line from a grade-1 meningioma of a patient with NF2-related schwannomatosis (NF2-SWN). We showed that like Ben-Men-1 cells, AG-NF2-Men cells were *NF2*-null, expressed several *NF2*-regulated receptor tyrosine kinases, and responded to their cognate ligands. We also found that brigatinib and INK128 alone inhibited AG-NF2-Men cell proliferation at IC_50_ values similar to those in Ben-Men-1 cells. Combining brigatinib with INK128 exhibited growth-inhibitory synergy. Mechanistically, the combination not only completely abrogated p-AKT(S473) and its downstream signaling compared with either drug alone but also prevented INK128-mediated rephosphorylation of AKT on T308. Also, the combination more effectively blocked ligand-mediated phosphorylation of EGFR, ErbB3, and IGF-1R and elicited major changes in the expression of genes, including the upstream regulators of several signaling networks important for meningioma growth. Furthermore, we generated luciferase-expressing AG-NF2-Men cells that readily grew as intracranial xenografts. Importantly, combining brigatinib with INK128 enhanced tumor regression in both the orthotopic AG-NF2-Men and Ben-Men-1 xenograft models. As the first NF2-SWN–related meningioma cell line, AG-NF2-Men is a unique reagent for investigating meningioma biology and therapeutics. A clinical trial to evaluate the combination of brigatinib with an mTOR inhibitor in *NF2*-deficient meningiomas is warranted.

**Significance:**

AG-NF2-Men represents the first NF2-SWN–related meningioma model. The brigatinib + INK128 combination exhibits antitumor synergy in both the AG-NF2-Men and Ben-Men-1 meningioma models, suggesting combining brigatinib with mTOR inhibition to more effectively treat NF2-SWN and sporadic *NF2*-deficient meningiomas.

## Introduction

Constituting ∼40% of primary intracranial tumors, meningiomas, which originate from meningothelial cells of the arachnoid layer lining the brain and spinal cord, are the most common brain tumors (1). These tumors can arise at the convexity, the skull base, and along the spine and may occur at any age, but the incidence increases progressively with age (2). The majority (∼80%) of meningiomas are histologically benign [World Health Organization (WHO) grade 1], whereas the remaining are atypical (grade 2) and anaplastic (grade 3). Benign meningiomas may grow over years without causing symptoms. However, depending on the size and location of growth, they can cause significant morbidity, including seizures, vision loss, neurologic deficits, cranial nerve palsy, speech dysfunction, and motor weakness (3). Despite their high prevalence, there is presently no FDA-approved medical therapy.

Whereas most meningiomas occur sporadically, these tumors are frequently found in patients with neurofibromatosis type 2–related schwannomatosis (NF2-SWN), a highly-debilitating tumor-suppressor syndrome in which affected individuals develop multiple nervous system tumors, including meningiomas (4). Currently, surgical resection is the primary treatment strategy for symptomatic or rapidly growing meningiomas. However, surgical removal of meningiomas can damage adjacent brain tissue and cause additional neurologic deficits. Patients with NF2-SWN frequently develop multiple meningiomas. Surgical excision in these patients is often difficult, especially for tumors located along the skull base. Radiotherapy may be used to manage inoperable or recurrent cases, but radiation treatment increases the risk of malignant transformation or secondary cancers (5–7). These drawbacks in the current standard-of-care underscore the importance of identifying an effective medical therapy that stops tumor growth or eliminates meningiomas.

NF2-SWN is caused by biallelic inactivation of the *NF2* gene, which encodes the tumor-suppressor protein merlin that acts as a linker between the plasma membrane and actin cytoskeleton (8–10). Intriguingly, ∼50 to 60% of sporadic meningiomas also harbor *NF2* mutations, suggesting an important role for *NF2* loss in tumorigenesis. Merlin interacts with adherens junction components, mediating contact inhibition of cell proliferation (11). It blocks ligand-mediated internalization of the epidermal growth factor receptor (EGFR)/ErbB family members, other receptor tyrosine kinases (RTK), such as the insulin-like factor-1 receptor (IGF-1R) and platelet-derived growth factor receptor-α, and membrane-tethered non-RTKs like focal adhesion kinase (FAK; refs. 12–14). It may also inhibit the delivery of membrane receptors back to the plasma membrane. Furthermore, merlin can restrain the mitogen-activated protein kinase, phosphoinositide 3-kinase/AKT/mammalian target of rapamycin (mTOR), FAK-Src, and Hippo signaling pathways (15). In addition, merlin can suppress activation of the p21-activated kinase (PAK), and reciprocally, PAK can phosphorylate and inactivate merlin (16). These results suggest that loss of merlin disrupts multiple signaling pathways, leading to tumorigenesis, and that these pathways are potential therapeutic targets.

To identify an effective therapy for *NF2*-deficient meningioma, we (17) previously showed that the Ben-Men-1 cell line established from a sporadic grade-1 meningioma (18) is *NF2*-null. By generating luciferase-expressing Ben-Men-1-LucB cells and stereotactically injecting them into the skull base of immunodeficient mice, followed by bioluminescence imaging (BLI), we showed that Ben-Men-1-LucB cells established intracranial xenografts that grew slowly over time and did not invade the brain. As a member of the Synodos for NF2 consortium, we used this model to identify several targeted drugs that potently inhibited meningioma growth (19–21). We found that brigatinib (ALUNBRIG), originally designed as an anaplastic lymphoma kinase inhibitor (22), caused tumor shrinkage via inhibiting multiple RTKs and non-RTKs, including FAK (21). A phase II clinical trial of brigatinib in NF2-SWN patients with progressive tumors (NCT04374305) confirmed the greatest benefit of brigatinib for meningioma and nonvestibular schwannoma (23, 24). Also, we demonstrated that the dual mTORC1/2 inhibitor INK128 (sapanisertib) elicited strong suppression of meningioma growth (20).

Whereas Ben-Men-1 represents the first *NF2*-deficient meningioma cell line immortalized by only using telomerase, it was derived from a sporadic tumor (18). Curiously, compared with sporadic low-grade meningiomas, tumors in patients with NF2-SWN often exhibit more aggressive growth behavior and are more likely to become symptomatic and recur after resection (5, 6). Presently, the biology behind these differences is not understood. Also, it is not known whether sporadic and NF2-SWN–related meningiomas respond differently to therapies. The availability of a meningioma cell line established from an NF2-SWN tumor should allow us to compare their tumor biology and enhance NF2 therapeutic development.

In this study, we report the generation of the first telomerase-immortalized NF2-SWN–related meningioma cell line AG-NF2-Men. As single-agent brigatinib and INK128 effectively block the growth of *NF2*-deficient tumors (20, 21), we explored the possible antitumor synergy of combining these two targeted agents. We showed that in both the AG-NF2-Men and Ben-Men-1 models, the brigatinib + INK128 combination exhibited growth-inhibitory synergy by more effectively suppressing RTK-mediated phosphorylation of AKT and the mTOR target 4EBP1 (eIF4E-binding protein 1), as well as by eliciting major changes in the expression of genes including the upstream regulators of several signaling networks important for meningioma growth. We also generated luciferase-expressing AG-NF2-Men-Luc2 cells and showed that they readily established intracranial tumor xenografts in immunodeficient mice. Using both the intracranial AG-NF2-Men-Luc2 and Ben-Men-1-LucB meningioma models, we demonstrated that the brigatinib + INK128 combination enhanced antitumor effects.

## Materials and Methods

### Tumor procurement, immunohistochemistry, and FoundationOne test

A meningioma appearing as a firm, light tan-to-brown, fibrous mass (2 × 1.2 × 0.8 cm) was resected from the left cerebellopontine angle region of a 30-year-old male NF2-SWN patient with written informed consent according to the Ohio State University Institutional Review Board–approved Human Subjects protocol, and the studies were conducted in accordance with the US Common Rule. A piece of tumor was processed for paraffin sections, hematoxylin–eosin staining, and immunohistochemistry (IHC; see Supplementary Methods). A certified neuropathologist diagnosed the tumor as a WHO grade-1 meningioma. To detect genomic alterations, tumor sections were submitted to Foundation Medicine for a FoundationOne Heme next-generation sequencing test, which interrogates 406 genes commonly mutated in human cancers and selected introns of 31 genes involved in rearrangements, in addition to RNA sequencing (RNA-seq) of 265 genes.

### Immortalization, telomerase assay, and fluorescence *in situ* hybridization

A piece of freshly resected NF2-SWN meningioma tissue was used to prepare primary cell culture (see Supplementary Methods for details; ref. 25). Briefly, meningioma tissue was minced with scalpels, dissociated with collagenase and dispase, and then triturated to release tumor cells. Dissociated meningioma cells were grown in Dulbecco’s Modified Eagle Medium plus 10% fetal bovine serum (R&D Systems). Upon reaching confluence, primary meningioma cells were split into two dishes. The next day, one dish was infected with human telomerase reverse transcriptase (hTERT)-expressing retroviruses, which also carry a neomycin-resistant gene, in the presence of 8 mg/mL polybrene, and the other dish was incubated only with polybrene (MilliporeSigma). Following overnight incubation, the culture medium was replaced, and both dishes of cells were grown and passaged in medium without G418 selection until immortalized cells emerged and nontransduced cells senesced.

Telomerase enzymatic activities were measured using a telomeric repeat amplification protocol (TRAP) assay (Telomerase Activity Quantification qPCR Assay Kit; ScienCell). Briefly, telomerase in cell extracts catalytically adds telomeric repeats to a DNA substrate. The telomerase-elongated products are then amplified and detected by qPCR. Fluorescence *in situ* hybridization (FISH) analysis was performed using the Vysis EWSR1 (22q12) Dual Color Break Apart Rearrangement FISH Probe Kit (Abbott Molecular) on nuclei from meningioma cells (Supplementary Methods). Dual-color probe-hybridized cells were assessed by a board-certified cytogeneticist.

### Resazurin assays, drug combination matrix arrays, and cell counting

Cell proliferation of AG-NF2-Men cells with or without brigatinib or INK128 treatment was determined after 3 days by measuring the fluorescent readout of resazurin assays, and IC_50_ (50% inhibitory concentration) values were estimated using GraphPad Prism v10 (17). For drug combinations, AG-NF2-Men and Ben-Men-1 cells seeded at 4,000 and 2,000 cells/well, respectively, as 8 × 8 matrices in 96-well plates were treated with brigatinib and INK128 arrays of a 7-point, 2-fold dilution series. Normalized cell proliferation was acquired by resazurin assays, and synergy scores were calculated using the Loewe additivity model in SynergyFinder v3.0 (26). In addition, the growth-inhibitory synergy of the brigatinib + INK128 combination in AG-NF2-Men cells was assessed by cell counting. For details, please see Supplementary Methods.

### Incucyte live cell imaging of apoptosis, Western blots, and immunofluorescence staining

These techniques were performed according to Supplementary Methods.

### RNA-seq analysis

AG-NF2-Men cells were treated in triplicate with 1 x IC_50_ of brigatinib, INK128, brigatinib + INK128, or an equivalent amount of DMSO (0.01%). After 24-hour incubation, cell pellets were harvested and submitted to MedGenome for RNA-seq (Supplementary Methods).

### Generation of luciferase-expressing AG-NF2-Men cells and meningioma xenograft models

Immortalized AG-NF2-Men cells were infected with luciferase-expressing lentiviruses (17). The clone expressing the highest luciferase activity, designated AG-NF2-Men-Luc2, was tested for its ability to establish xenografts by injecting 5 × 10^5^ cells subcutaneously in the flank or stereotactically into the skull base of 8- to 12-week-old NSG mice (The Jackson Laboratory). All animal procedures were performed according to the protocol approved by our Institutional Animal Care and Use Committee. Injected mice were monitored by weekly BLI. Mice with established tumors were randomized into different treatment groups and received vehicle, 50 mg/kg of brigatinib, 0.75 mg/kg of INK128, or their combination (*n* = 10/group) every day by oral gavage (20, 21). The effects of treatment were monitored by weekly BLI. Similarly, we generated mice bearing intracranial Ben-Men-1-LucB xenografts for drug treatments (17). Pairwise comparison statistical analysis was performed using the TumGrowth website (https://kroemerlab.shinyapps.io/TumGrowth/), with *P* values calculated using cross-sectional analysis of log-transformed normalized tumor luminescence.

## Results

### Establishment of the *bona fide* NF2-SWN meningioma cell line AG-NF2-Men

**Figure 1.**
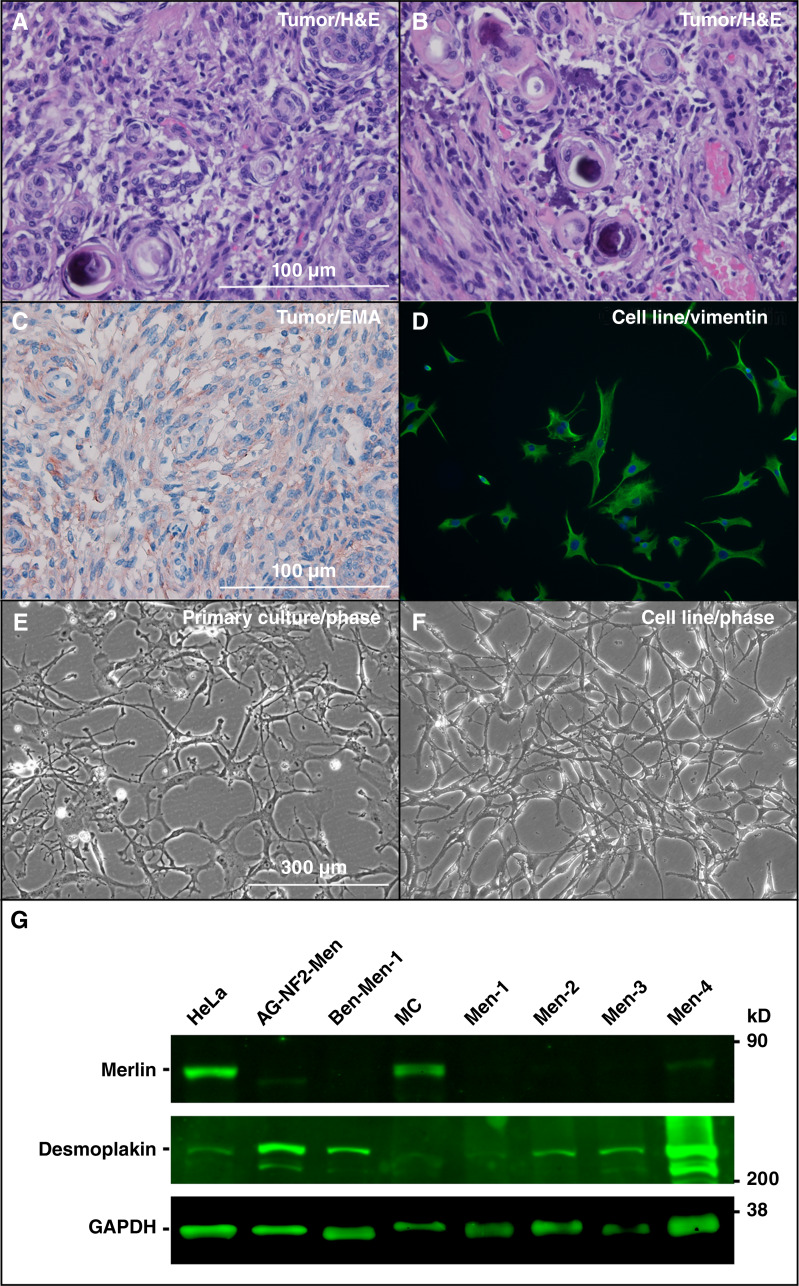
Morphologic and immunologic characterization of the AG-NF2-Men tumor and immortalized cell line. **A** and **B,** Hematoxylin and eosin (H&E) staining of tumor sections of the AG-NF2-Men meningioma from an NF2-SWN patient showed a typical whorling architecture and psammoma bodies. **C,** IHC analysis of AG-NF2-Men tumor sections revealed membrane and cytoplasmic expression of EMA, commonly found in meningioma cells. **D,** Immunofluorescence staining detected VIM expression (green) in the cytoplasm of AG-NF2-Men cells. DAPI stained the nuclei in blue. **E** and **F,** Phase-contrast micrographs of the AG-NF2-Men primary culture (**E**) and immortalized cells (**F**). Like the primary culture, immortalized AG-NF2-Men cells exhibit a spider web–like growth pattern. **G,** Western blot analysis confirmed that like the Ben-Men-1 cell line established from a sporadic grade 1 meningioma, AG-NF2-Men cells did not express detectable merlin protein, whereas normal meningeal cells (MC) and HeLa cervical carcinoma cells readily expressed merlin. Also, the merlin protein was not detected in primary cultures of four meningiomas from different patients (Men-1 to Men-4). In addition, DSP, a desmosomal component often expressed by meningioma cells, was detected in AG-NF2-Men cells, Ben-Men-1 cells, MCs, and primary meningioma cells. kD, kilodalton of molecular weight.

To generate an NF2-SWN meningioma cell line, we procured a WHO grade-1 meningioma from an NF2-SWN patient. Histologically, the tumor contained meningothelial cells appearing relatively large and elongated with a whorling architecture and psammoma bodies (Fig. 1A and B) and expressed the meningioma marker epithelial membrane antigen (EMA; Fig. 1C). There were no atypia or atypical mitosis and no evidence of malignancy. Despite the patient having previously received several experimental drug treatments, the tumor exhibited low mutational burden and stable microsatellite status with only two pathogenic variants identified: a splice site mutation (447+1G>C) in the *NF2* gene and a duplication of *MYC* exons 2 to 3. Also, the tumor expressed several phosphorylated merlin-regulated kinases, including ErbB3 and FAK, and their downstream signaling molecules AKT and ERK1/2, compared with normal human sciatic nerves and mouse brain and meninge tissues (Supplementary Fig. S1A–S1L). Additionally, the tumor had strong nuclear labeling of cMYC (Supplementary Fig. S1M–S1O) and abundant macrophages that were immunopositive for CD163, a marker of M2 macrophages that often comprise protumorigenic microenvironments (Supplementary Fig. S1P–S1R).

Next, we prepared primary cell culture from this NF2-SWN meningioma and transduced it with hTERT-expressing retroviruses, which also carry a neomycin-resistant gene. To enhance propagation of immortalized cells, transduced cells were grown and split in growth medium without G418 selection until immortalized cells emerged. We reasoned that as primary *NF2*-deficient tumor cells have poor transduction efficiency and do not grow well at low density, G418 selection immediately following retrovirus infection would eliminate untransduced cells, leaving only few telomerase-transduced cells that might not grow in sparse conditions. Primary meningioma cells usually grow in culture for only 10 to 15 passages before they senesce. However, without G418 selection, we observed that after 15 passages, some cells in the telomerase-transduced dish continued to grow and formed small colonies surrounded by residual enlarged, flattened, and vacuolating senesced cells. More colonies or islands of cells were seen in subsequent passages, suggesting possible immortalization. Therefore, we added G418 to the culture at passage 18 and found that these colonies could grow to confluence. These immortalized cells, designated AG-NF2-Men, were subcloned and have been grown for more than 100 passages.

### The AG-NF2-Men cell line is *NF2*/merlin-null and exhibits characteristics of a benign meningioma

To verify that immortalized AG-NF2-Men cells were derived from the tumor cells in the original NF2-SWN–related meningioma, we first performed FISH analysis to determine the status of chromosome 22 in the primary culture and immortalized cells. Using dual-color DNA probes covering the chromosome 22q12 region in which the *NF2* gene is located, we found that the majority of cells in the primary meningioma culture exhibited only one red-green signal, indicating the presence of only one copy of chromosome 22 (Supplementary Fig. S2A and S2B). The primary culture also showed sporadic instances of cells with two copies of chromosome 22 (Supplementary Fig. S2B), which are likely contaminating stromal cells present in the primary culture. Importantly, immortalized AG-NF2-Men cells displayed uniform chromosome 22 monosomy (Supplementary Fig. S1C). Together with the mutational analysis from FoundationOne test, these results suggest that AG-NF2-Men tumor cells have *NF2* mutations on one chromosome 22 and a loss of the other chromosome 22.

Immortalized AG-NF2-Men cells exhibited relatively slow growth with a doubling time of ∼2.6 days (Supplementary Fig. S3), similar to that of Ben-Men-1 cells but slower than that of *NF2*-deficient KT21-MG1 malignant meningioma cells, which have a 1-day doubling time (17, 27). Morphologically, immortalized AG-NF2-Men cells seemed multi-polar with numerous processes resembling lamellipodia and filopodia, characteristic of NF2-SWN-related tumor cells (28, 29). Similar to the primary AG-NF2-Men culture, they grew in a spider web pattern reminiscent of leptomeningeal cells (Fig. 1E and F), indicating that telomerase immortalization did not alter cell morphology. RNA-seq analysis detected expression for desmoplakin (*DSP*), other desmosomal proteins desmoglein 2 (*DSG2*), desmocollin 3, and plakophilin 2 (*PKP2*) and *PKP4*, as well as *EMA* and vimentin (*VIM*) in immortalized AG-NF2-Men cells (Supplementary Fig. S4). Consistently, we detected the expression of VIM and DSP 1/2 proteins (Fig. 1D and G), which are characteristically co-expressed by arachnoidal and meningioma cells (30, 31). However, AG-NF2-Men did not express DSG3 RNA (Supplementary Fig. S4) and protein as has been reported in meningiomas (30). In line with the FoundationOne and FISH tests, AG-NF2-Men cells did not have any merlin protein (Fig. 1G) but expressed several merlin-regulated EGFR, ErbB3, and IGF-1R. These RTKs underwent robust ligand-mediated autophosphorylation, followed by downstream activation of p-AKT and p-ERK1/2 (Supplementary Fig. S5). RNA-seq analysis of RTK:ligand pairs showed that AG-NF2-Men cells expressed a low level of NRG1, which codes for the ErbB3 ligand neuregulin/heregulin but not EGF and IGF-1 (Supplementary Fig. S4B).

**Figure 2.**
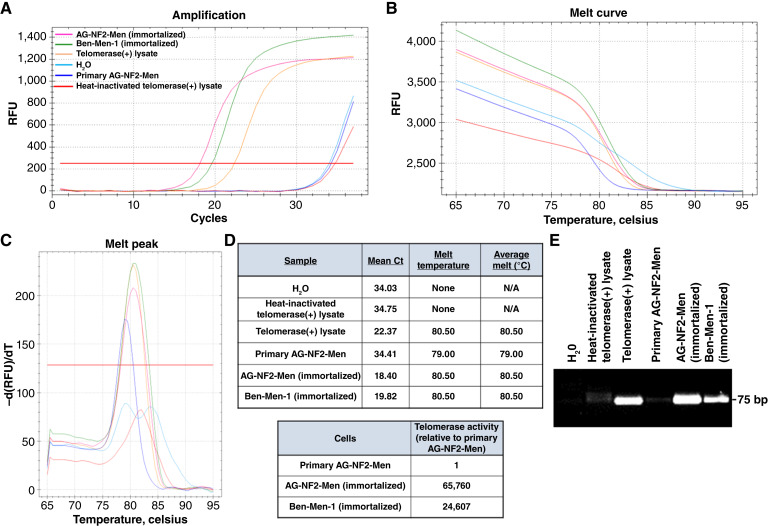
Immortalized AG-NF2-Men cells demonstrated robust telomerase activity. **A,** Real-time PCR amplification curves of a telomerase-specific template after incubation with cell extracts from primary AG-NF2-Men cells and telomerase-immortalized AG-NF2-Men and Ben-Men-1 cells. Manufacturer-supplied lysates from telomerase-expressing cells before and after heat inactivation served as positive and negative assay controls, respectively. A no-template control (H_2_O) was used as an additional negative control. The horizontal red line indicates the threshold at which the C_t_ values were measured. **B** and **C,** Melt curve (**B**) and Melt peak (**C**) analysis of the telomeric repeat amplicons at the end of the PCR analysis. **D,** Tables summarizing the mean Ct and melt temperatures of the amplicons and the estimated fold increase in telomerase activity in immortalized AG-NF2-Men and Ben-Men-1 cells vs. primary AG-NF2-Men cells. The telomerase activity in immortalized AG-NF2-Men cells was ∼6.6 × 10^4^-fold higher than that in the primary culture. **E,** An agarose gel showing that high levels of telomerase-mediated elongation of the synthetic telomeric repeat template occurs only in telomerase-immortalized AG-NF2-Men and Ben-Men-1 cells.

To verify human telomerase expression, we performed the TRAP, which directly measures telomerase enzymatic activity. Primary nontransduced AG-NF2-Men cells at passage 2 had little or no telomerase activity (the cycle threshold value C_t_ = 34.41), similar to the negative control with H_2_O (C_t_ = 34.03) or heat-inactivated telomerase (+) cell lysate (C_t_ = 34.75; Fig. 2A–D). In contrast, immortalized AG-NF2-Men cells at passage 37 had robust telomerase activity, comparable with the Ben-Men-1 cell line (C_t_ = 18.40 vs. 19.82, respectively). Melt-curve analysis of the telomerase reactions from immortalized AG-NF2-Men and Ben-Men-1 cells showed a single peak with a melting temperature nearly identical to the positive control, and gel electrophoresis confirmed the presence of the telomerase amplicon in immortalized AG-NF2-Men and Ben-Men-1 cells (Fig. 2B, C, and E). These results indicate that immortalized AG-NF2-Men cells stably express high levels of telomerase activity.

Together, these results indicated the establishment of an NF2-SWN–related meningioma cell line that retains characteristics of a benign meningioma and expresses several RTKs implicated in driving meningioma growth.

### INK128 synergizes with brigatinib to block AG-NF2-Men cell proliferation by completely abrogating AKT and 4EBP1 phosphorylation

**Figure 3.**
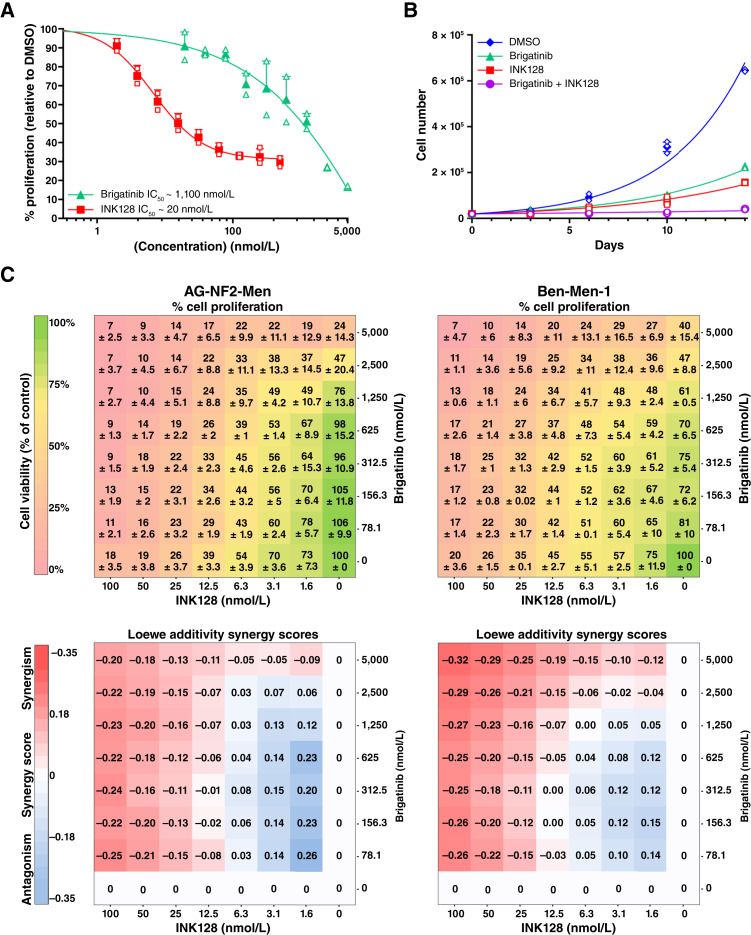
The brigatinib + INK128 combination exhibited synergistic antiproliferative activity in AG-NF2-Men cells. **A,** Dose–response curves from proliferation assays of AG-NF2-Men cells treated for 3 days with serial dilutions of each indicated drug established a mean IC_50_ of 1,100 nmol/L for brigatinib and 20 nmol/L for INK128. **B,** AG-NF2-Men cells were treated for up to 14 days with DMSO or 1 x IC_50_ of brigatinib or INK128 alone or in combination. Cells were harvested in biological duplicates and counted using a hemocytometer. Solid symbols and error bars for both graphs in (**A**) and (**B**) represent the mean + SD from two independent experiments. The open symbols show the percent growth inhibition (**A**) and cell numbers (**B**) from each independent experiment. **C,** AG-NF2-Men (left) and Ben-Men-1 (right) cells were treated in a combinatorial array with brigatinib and INK128, and cell proliferation was measured relative to DMSO control cells designated 100%. Shown above are the proliferation matrices indicating the means + SEs from three (AG-NF2-Men) and two (Ben-Men-1) independent experiments. Loewe additivity synergy score matrices are depicted below. Blue shading denotes drug antagonism, whereas red shading indicates enhanced growth inhibition by the brigatinib + INK128 combination. Loewe scores ≤ −0.1 signify synergistic drug interaction.

As loss of *NF2* function is associated with deregulated mTORC1/2 signaling (32, 33), we previously showed that the dual mTORC1/2 inhibitor INK128 effectively suppresses the growth of Ben-Men-1 cells and intracranial xenografts (20). Also, the multikinase inhibitor brigatinib causes tumor shrinkage (21). However, brigatinib alone did not eliminate all Ben-Men-1 cells as treated tumors resumed growth upon cessation of treatment. Therefore, we examined whether combining INK128 with brigatinib would enhance antitumor effects in both AG-NF2-Men and Ben-Men-1 models. Brigatinib and INK128 elicited growth inhibition in AG-NF2-Men cells, with the IC_50_ values of ∼1.1 μmol/L and 20 nmol/L, respectively (Fig. 3A), similar to those in Ben-Men-1 cells (21, 34). To examine whether the brigatinib + INK128 combination exhibited growth-inhibitory synergy, we first performed cell counting assays. Whereas brigatinib or INK128 alone substantially reduced the growth of AG-NF2-Men cells, combined brigatinib + INK128 treatment almost completely arrested their growth (Fig. 3B). We next treated both AG-NF2-Men and Ben-Men-1 cells with brigatinib and INK128 in 8 × 8 drug matrix arrays and measured the effects on cell proliferation. The brigatinib + INK128 combination synergistically inhibited proliferation of both meningioma cell lines with maximum Loewe synergistic scores of −0.25 and −0.31 for AG-NF2-Men and Ben-Men-1 cells, respectively (Fig. 3C).

**Figure 4.**
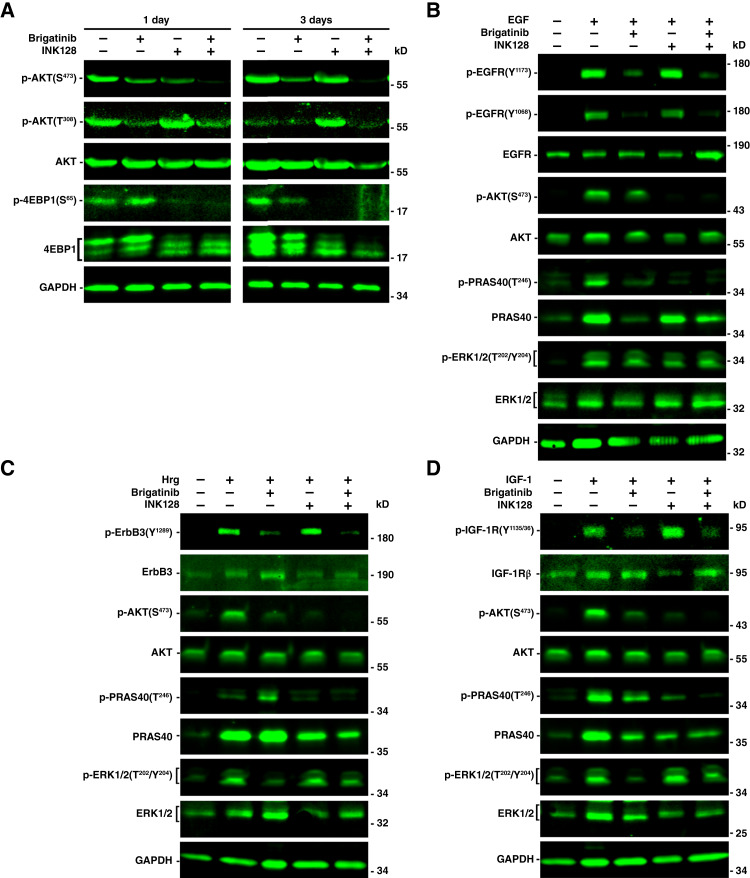
The brigatinib + INK128 combination elicited a superior and more durable suppression of AKT and 4EBP1 phosphorylation compared with the individual drugs. **A,** AG-NF2-Men cells were treated for 1 and 3 days with DMSO vehicle or 1 x IC_50_ dose of brigatinib (1.1 μmol/L) or INK128 (20 nmol/L) as single agents or in combination. Treated cell lysates were subjected to Western blot analysis for total and phosphorylated AKT and the mTORC1 target and protein translation repressor 4EBP1. GAPDH served as the loading control. **B–D,** AG-NF2-Men cells were serum-starved for 24 hours to induce quiescence and then treated with brigatinib, INK128, or brigatinib + INK128 in serum-free medium for 2 hours, followed by growth stimulation for 5 minutes with 50 ng/mL EGF (**B**), Hrg (**C**), or IGF-1 (**D**). Lysates from these cells were analyzed by Western blotting for ligand-mediated phosphorylation of their cognate RTKs and downstream phosphorylation and activation of AKT/PRAS40 and ERK1/2. GAPDH serves as the loading control. kD, kilodalton of molecular weight.

Brigatinib treatment partially reduced p-AKT on both S473 and T308 in AG-NF2-Men cells after 1-day treatment, and this partial reduction in p-AKT(S473) and p-AKT(T308) was still observed after 3 days (Fig. 4A). In contrast, INK128 treatment for 1 day only reduced p-AKT(S473), but not p-AKT(T308), and this effect was transient as the p-AKT(S473) level almost returned to the baseline level by 3 days. Additionally, INK128 alone slightly increased p-AKT(T308) after 1-day treatment, and the increase in p-AKT(T308) was even more pronounced after 3 days, suggesting activation of feedback signaling leading to rephosphorylation of AKT at this residue. As 4EBP1 is a major target of the AKT/mTORC1 pathway and function as a translational repressor that is inactivated after phosphorylation by these kinases, we found that INK128 reduced p-4EBP1 at S65, a key phosphorylation site for regulating its binding to eIF4E (35). Consistently, an increase in faster-migrating, hypophosphorylated species of 4EBP1 was observed after INK128 treatment. However, brigatinib did not affect the p-EBP1(S65) level after 1-day treatment and only moderately decreased p-4EBP(S65) after 3 days. Thus, when the blots were probed for total 4EBP1, abundant slow-migrating, hyperphosphorylated 4EBP1 species were detected in brigatinib-treated AG-NF2-Men cells after 3 days, similar to DMSO-treated controls. Importantly, the brigatinib + INK128 combination not only more effectively blocked p-AKT(S473) at both time points than either drug alone but also completely prevented INK128-mediated rephosphorylation of AKT on T308 by 3 days. Also, the combination diminished p-4EBP1(S65) after 1-day treatment. Whereas brigatinib alone only slightly reduced p-4EBP1, combined brigatinib + INK128 treatment effectively converted the majority of 4EBP1 to a fast-migrating, hypophosphorylated species.

Previously, we showed that brigatinib inhibits multiple merlin-regulated RTKs, e.g., EGFR, ErbB3, and IGF-1R, in Ben-Men-1 cells (21). Therefore, we investigated the ability of the brigatinib + INK128 combination to suppress ligand-mediated growth signaling from these RTKs. AG-NF2-Men cells, when serum-starved for 24 hours, did not express p-EGFR at its autophosphorylation sites Y1173 and Y1068 and exhibited little or no p-AKT(S473) and its downstream p-PRAS40, as well as p-ERK1/2 (Supplementary Fig. S6). Whereas brigatinib treatment did not affect phosphorylation of these signaling molecules in growth-arrested AG-NF2-Men cells, INK128 alone and its combination with brigatinib seemed to slightly enhance p-ERK1/2, consistent with previous findings indicating compensatory phosphorylation and activation of ERK1/2 signaling by AKT/mTOR inhibition. Addition of EGF rapidly induced p-EGFR(Y1173 and Y1068), p-AKT(S473), p-PRAS40, and p-ERK1/2 (Fig. 4B). Brigatinib treatment completely blocked ligand-mediated phosphorylation of EGFR at Y^1068^. Intriguingly, brigatinib substantially reduced but did not eliminate p-EGFR(Y1173), and as a result, some p-AKT and p-ERK1/2 were still detected in treated cells. INK128 treatment did not affect EGFR phosphorylation but completely blocked p-AKT and its downstream p-PRAS40. The brigatinib + INK128 combination effectively suppressed p-EGFR, p-AKT, and p-PRAS40. Like brigatinib alone, the combination-treated cells still exhibited a small but detectable level of p-EGFR(Y1173) and p-ERK1/2 in EGF-stimulated cells. Similar results were observed for ErbB3, IGF-1R, AKT, and PRAS40 phosphorylation in Hrg-stimulated and IGF-1–stimulated AG-NF2-Men cells (Fig. 4C and D). In particular, the brigatinib + INK128 combination had greater efficacy at diminishing p-AKT and p-PRAS40 compared with brigatinib alone and more effectively reduced ligand-mediated receptor phosphorylation compared with the INK128 treatment. Interestingly, we observed that whereas single-agent brigatinib or INK128 had little caspase activation similar to the DMSO controls, the brigatinib + INK128 combination elicited an increase in cleaved caspase positive labeling (Supplementary Fig. S7), consistent with a decrease in prosurvival signaling from p-RTKs and p-AKT.

Collectively, these results suggest that INK128 enhances the ability of brigatinib to suppress RTK-mediated AKT phosphorylation at S473, prevents feedback rephosphorylation at T308, and blocks 4EBP1 phosphorylation, leading to synergistic growth inhibition.

### Combining INK128 with brigatinib elicits an exaggerated transcriptomic response with suppression of multiple drivers of meningioma growth

**Figure 5.**
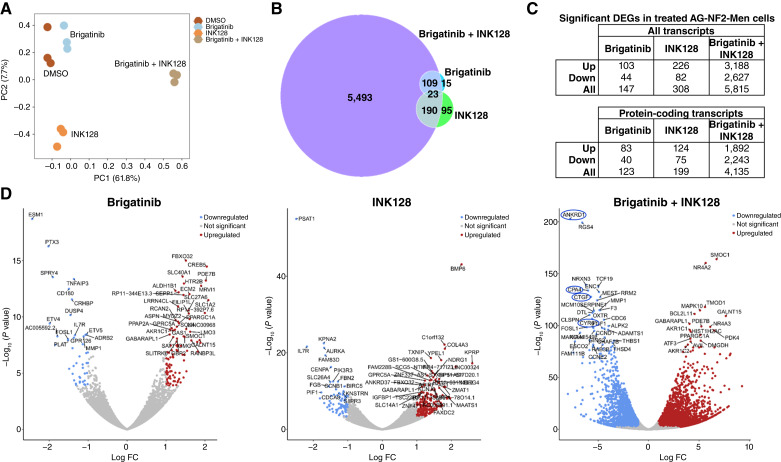
AG-NF2-Men cells treated with the brigatinib + INK128 combination exhibited profoundly altered gene expression programs associated with growth suppression, including reduced expression of YAP target genes. **A,** Principal component analysis demonstrated that the biological triplicates from each treatment group clustered together. Groups separated by drug treatment are DMSO (brown), brigatinib (light blue), INK128 (orange), and brigatinib + INK128 (beige). **B,** Venn diagram showing that the brigatinib + INK128 combination–treated AG-NF2-Men cells exhibited 5,493 significant unique DEGs [P adj ≤ 0.01; antibodies (log_2_ FC) ≥ 1], whereas single agents only elicited a small number of DEGs. **C,** Tables showing a substantially larger number of upregulated and downregulated DEGs of all types (upper table) and of protein-coding genes (lower table) in the combination-treated cells compared with individual drug treatments. **D,** Volcano plots showing DEGs in AG-NF2-Men cells treated with brigatinib (left), INK128 (middle), and the brigatinib + INK128 combination (right) relative to the DMSO-treated controls. Downregulated genes are colored blue and upregulated genes are red, with the top 50 DEGs labeled. The YAP target genes *ANKRD1*, *CTGF*, *CPA4*, and *CYR61* are circled. FC, fold change.

To further investigate the mechanism underlying the enhanced antiproliferative effects of the brigatinib + INK128 combination, we performed RNA-seq analysis on various drug-treated AG-NF-Men cells. Principal component analysis showed clear separation by drug treatment (Fig. 5A). Single-agent brigatinib and INK128 only modestly affected gene expression with only 147 and 308 differentially expressed genes (DEG), respectively (Fig. 5B and C; Supplementary Data S1). However, the brigatinib + INK128 combination treatment significantly affected the expression of 5,815 genes, with 5,493 DEGs exclusive to the combination treatment. Among the top 50 DEGs, the brigatinib + INK128 combination significantly decreased transcripts encoding proteins important for DNA synthesis and cell-cycle progression, e.g., *MCM10*, *CCNE2*, and *CCND1* (Fig. 5D). Interestingly, four of the top 50 DEGs were the YAP target genes, including *ANKRD1*, *CPA4*, *CTGF*, and *CYR61*, and their expression was strongly decreased in combination-treated cells (Fig. 5D; Supplementary Fig. S8). Also, the expression of several other YAP-regulated genes, e.g., *AMOTL2*, *AXL*, *ANXA3*, *AJUBA*, *ANXA1*, and *CITED2*, were reduced, whereas single-agent brigatinib and INK128 did not significantly affect the expression of these YAP target genes (*P* adj ≤ 0.01).

IPA showed that only the combination-treated AG-NF2-Men cells were predicted to have statistically-significant inhibition of EGFR, ErbB3, ErbB4, and NRG1 signaling (Supplementary Fig. S9; Supplementary Data S2). These findings are consistent with the inhibition of ligand-induced signaling from EGFR and ErbB3 in AG-NF2-Men cells treated with brigatinib and its combination with INK128 (Fig. 4). Additionally, activation of AKT, an important mitogenic downstream target of the ErbB family and mTOR, was predicted to be suppressed. Consistent with the reduced transcript levels of multiple YAP transcriptional targets (Fig. 5D; Supplementary Fig. S8), the transcriptional activities of YAP, TAZ, and TEAD were predicted to be reduced. Concomitantly, LATS1 and LATS2, which repress YAP, were predicted to be activated.

Together, our results demonstrate that the brigatinib + INK128 combination more durably suppresses the downstream signaling of their kinase targets and the activity of a broad range of mitogenic signaling molecules, leading to growth-inhibitory synergy in treated AG-NF2-Men cells.

### The brigatinib + INK128 combination elicits enhanced tumor suppression in mice bearing intracranial AG-NF2-Men-Luc2 or Ben-Men-1-LucB xenografts

To establish a quantifiable animal model for NF2-SWN–associated meningioma, we generated luciferase-expressing AG-NF2-Men-Luc2 cells and injected them subcutaneously in the back or orthotopically in the skull base of NSG mice. Intriguingly, BLI showed that AG-NF2-Men-Luc2 cells did not grow when implanted at the subcutaneous location, whereas intracranially implanted xenografts readily grew over time (Supplementary Fig. S10). These results indicated that the tumor microenvironment (TME) is critical in supporting meningioma growth.

**Figure 6.**
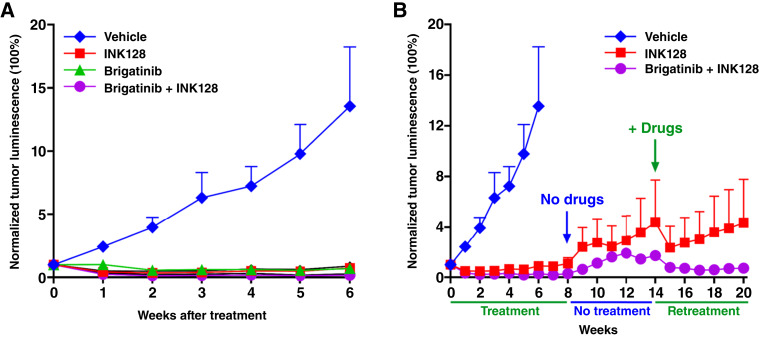
The brigatinib + INK128 combination effectively shrank intracranial AG-NF2-Men-Luc2 meningioma xenografts. **A,** Mice with established meningioma xenografts were treated with vehicle, brigatinib, INK128, or the brigatinib + INK128 combination by oral gavage, and tumor growth was monitored by BLI. The relative tumor-emitted BL signals were quantified and denoted as % of total flux after treatment relative to the total flux prior to treatment designated as one (100%). **B,** To assess potential tumor regrowth, we stopped treating mice in the INK128 or combination treatment group for 8 weeks and continued monitoring tumor growth by BLI for another 6 weeks. To examine the effects of re-treatment, we resumed treatment and continued BLI monitoring for 6 more weeks. The data are shown as the mean ± SE.

To examine the antitumor activities of brigatinib, INK128, and their combination, we generated and treated a cohort of mice bearing established intracranial AG-NF2-Men-Luc2 xenografts. As expected, vehicle-treated tumors grew over time with ∼13-fold increase of tumor-emitted bioluminescence over 6 weeks relative to pretreatment (Fig. 6A; Table 1). As in the Ben-Men-1-LucB model (21), brigatinib alone caused tumor shrinkage over 6-week treatment with the tumor-emitted bioluminescence signal reduced to ∼71% of pretreated tumors. INK128 alone also effectively blocked meningioma growth, and the bioluminescence signal emitted by the tumors treated for 6 weeks was reduced to ∼75% of pretreated tumors. Notably, combining INK128 with brigatinib elicited the strongest effect on tumor regression and greatly reduced tumor-emitted bioluminescence to ∼18% of pretreated tumors after 6 weeks (Table 1; Supplementary Fig. S11).

**Table 1.** The relative response of AG-NF2-Luc2 tumor xenografts in mice prior to and after treatment with vehicle, brigatinib, INK128, or brigatinib + INK128.

Week	Vehicle	SE	Brigatinib	SE	INK128	SE	Brigatinib + INK128	SE
0	1	0	1	0	1	0	1	0
1	2.43	0.33	1	0.07	0.42	0.08	0.24	0.06
2	3.95	0.80	0.57	0.03	0.35	0.10	0.14	0.05
3	6.30	2.01	0.62	0.06	0.38	0.08	0.15	0.06
4	7.23	1.55	0.61	0.08	0.50	0.10	0.18	0.08
5	9.78	2.32	0.56	0.19	0.48	0.09	0.14	0.06
6	13.55	4.69	0.71	0.01	0.75	0.19	0.18	0.11

The tumor-emitted luminescence for each indicated time point after treatment was normalized to that prior to treatment (week 0) designated as 1. Shown are the mean of relative tumor-emitted luminescence with SE.

To assess potential tumor regrowth after cessation of treatment, we stopped treating one cage of mice in the groups that had been treated with INK128 alone or the brigatinib + INK128 combination for 8 weeks and continued monitoring tumor growth by BLI for another 6 weeks. Upon treatment cessation, both INK128- and combination-treated tumors exhibited some regrowth; however, the combination-treated tumors regrew at much slower rates than the INK128-treated tumors (Fig. 6B). To examine the effects of retreatment, we resumed treatment of these mice for six more weeks. Encouragingly, tumor shrinkage was observed when the treatment was reinitiated. In particular, the regrown tumors shrank substantially upon retreatment with the brigatinib + INK128 combination, whereas the tumors retreated with INK128 alone showed only transient tumor shrinkage on the first week.

In addition, like those we reported previously (20, 21), brigatinib and INK128 alone blocked the growth of Ben-Men-1-LucB xenografts. Combining INK128 with brigatinib also enhanced tumor regression in this sporadic *NF2*-deficient meningioma model (Supplementary Fig. S12). Collectively, these results indicate that the brigatinib + INK128 combination elicits antitumor synergy against both NF2-SWN–related and sporadic *NF2*-deficient meningiomas.

## Discussion

To help identify novel medical therapies beneficial for patients with NF2-SWN–related meningiomas, it is important to use an animal model that recapitulates benign growth characteristics of these intracranial tumors. Genetically engineered mice with *Nf2* inactivation in leptomeningeal cells or prostaglandin D2 synthase–expressing meningeal progenitor cells develop meningiomas; however, the tumors take several months to develop, exhibit variable growth, and are detected in only a fraction of these mice (36, 37), limiting their use for therapeutic evaluation. Direct implantation of patient-derived meningioma tumor pieces into immunodeficient mice has been attempted. However, intracranial sites of inoculation may be challenging, and the growth of benign meningioma is limited (38, 39). Alternatively, primary human meningioma cells can be prepared to generate xenografts, but these primary cultures have limited lifespan. Several grade-1 meningioma cell lines have been generated, mostly by transfecting primary tumor cells with an oncogene or an oncogene plus telomerase; however, these oncogene-transformed cell lines exhibit altered growth signaling and behavior (31). Using only telomerase, Püttmann and colleagues (18) established the Ben-Men-1 cell line from a grade-1 meningioma. We showed that Ben-Men-1 is *NF2*-null (17). We also generated luciferase-expressing Ben-Men-1-LucB cells and used them to establish an orthotopic, quantifiable, *NF2*-deficient meningioma model. This model has facilitated identification of several potent targeted drugs (17, 20, 21, 34, 40). However, the Ben-Men-1 cell line was established from a sporadic tumor of a 68-year-old patient (18) and may not completely reflect the biology of NF2-SWN–related meningiomas, which often develop in younger patients (41). In addition to distinct molecular features, NF2-SWN–related meningiomas often exhibit more aggressive clinical behavior. Using a modified immortalization method in the present study, we generated the first telomerase-immortalized NF2-SWN-related meningioma cell line, AG-NF2-Men, from a grade-1 tumor of a young adult with NF2-SWN.

The original tumor from which the AG-NF2-Men cell line was derived carries low mutational burden (1 mutation/Mb). We identified mutations only in the *NF2* gene on chromosome 22 and the *MYC* gene on chromosome 8. FISH analysis confirmed the loss of one chromosome 22 in both the original tumor and immortalized AG-NF2-Men cells, suggesting the loss of heterozygosity in *NF2*. Consistently, the merlin protein was not detected in AG-NF2-Men cells. Like in *NF2*-deficient tumors, AG-NF2-Men cells expressed several merlin-regulated RTKs, including EGFR, ErbB3, and IGF-1R, and responded to their cognate ligands. Activation of these RTKs led to robust phosphorylation and activation of their downstream signaling molecules AKT and ERK1/2 to drive meningioma cell proliferation. Although the effect of duplication of *MYC* exons 2 and 3, which includes the entire cMYC protein-coding sequence (42), is not known, strong cMYC labeling was detected in the nucleus of AG-NF2-Men cells. In addition, AG-NF2-Men cells expressed several markers characteristic of meningiomas, e.g., DSP, VIM, and EMA. Our observation that the luciferase-expressing AG-NF2-Men-Luc2 cells could grow as intracranial but not subcutaneous xenografts reinforces the importance of the TME in supporting meningioma growth.

Studies have shown that *NF2* inactivation leads to dysregulation of multiple signaling pathways (6). As in the Ben-Men-1 model (20, 21), the multikinase inhibitor brigatinib and mTOR kinase inhibitor INK128 exhibited promising growth-inhibitory and antitumor activities in the AG-NF2-Men model, including comparable IC_50_ concentrations, reduced phosphorylation of EGFR, ErbB3, IGF-1R, and their downstream AKT and ERK1/2, as well as tumor shrinkage. Together with the clinical activity of brigatinib in NF2-SWN patients with meningiomas observed in the INTUITT-NF2 trial (23, 24), these results further support the extension of using brigatinib to treat sporadic meningiomas with pathogenic variants in the *NF2* gene. As NF2-related tumors frequently exhibit mTOR activation, mTOR inhibitors have also been investigated as potential treatments; however, first-generation rapamycin analogs, such as everolimus, show limited clinical efficacy, possibly because of incomplete inhibition of mTORC1 (43–46). Second-generation mTOR inhibitors, such as INK128 (sapanisertib) and AZD2014 (vistusertib), which directly block mTOR kinase activity, suppress both the mTORC1 and mTORC2 complexes. AZD2014 shows some clinical efficacy with stable disease in NF2 patients with progressive or symptomatic meningiomas, but unfortunately at the dose used, it was poorly tolerated (47), suggesting a need of dose reduction or combination with other targeted agents. Third-generation mTOR inhibitors, such as RMC-6272 and RMC-5552, were developed to ameliorate the poor tolerability of mTOR kinase inhibitors while maintaining their high potency (48). RMC-6272 elicits superior antiproliferative activity with durable inhibition of 4EBP1 phosphorylation in *NF2*-deficient meningioma cells, compared with first- and second-generation mTOR inhibitors (34). Also, RMC-6272 effectively blocks the growth of intracranial *NF2*-deficient meningioma xenografts.

It should be noted that although brigatinib acts as a multikinase inhibitor, its efficacy in our *NF2*-deficient meningioma models is likely mediated, at least in part, via inhibition of multiple RTKs, including ErbB3 and IGF-1R, and non-RTKs, such as FAK, important for driving meningioma growth (21). However, FAK inhibition alone had limited clinical activity in patients with *NF2*-mutated tumors, including meningiomas (48). Although ERBB3 inhibition partially downregulated mTOR pathway activation, it had no effect on the viability of *NF2*-deficient meningioma cells. Combined inhibition of mTOR and IGF-1R resulted in synergistic effects on cell viability (49). Given that brigatinib inhibits all these RTKs and additional kinases (21), it is expected that the brigatinib + INK128 combination elicit further antitumor effects against meningiomas.

NF2-SWN–related tumors exhibit heterogeneity with variable responses to treatment, suggesting a need for combination treatment. Using both the AG-NF2-Men and Ben-Men-1 models, we showed that the brigatinib + INK128 combination elicited antitumor synergy. Mechanistically, the combination produced a stronger suppression of p-AKT signaling and p-4EBP1, compared with individual agents. The abrogation of p-4EBP1 is particularly interesting as the ability to decrease p-4EBP1 correlates with the clinical efficacy of mTOR inhibitors (50). We also confirmed that INK128 likely induces feedback compensatory phosphorylation of p-AKT(T308) and p-ERK. Activated p-AKT and mTOR typically engage negative feedback inhibition pathways to prevent their unrestrained growth signaling. Drugs that interfere with this regulation can paradoxically promote mitogenic growth through compensatory activation of AKT and ERK1/2 (51). Thus, it is reassuring that the brigatinib + INK128 combination does not display reactivation of these molecules.

Transcriptomic analysis of drug-treated AG-NF2-Men cells revealed that single-agent brigatinib or INK128 affected the expression of only a limited number of genes. However, the brigatinib + INK128 combination–treated cells displayed a startling shift in their transcriptome, with more than 5,000 DEGs. Several of these DEGs correlated with inhibition of the EGFR/ErbB3 and AKT pathways, consistent with our findings from Western blotting. Surprisingly, several YAP-regulated genes were suppressed by the brigatinib + INK128 combination. As merlin negatively regulates the Hippo/YAP signaling (52), these results suggest that the combination broadly suppresses various mitogenic pathways deregulated in *NF2*-deficient meningioma cells. It will be interesting to see whether the combination inhibits YAP transcriptional activity, and, if so, the mechanisms by which it occurs.

Importantly, the brigatinib + INK128 combination showed enhanced tumor shrinkage of intracranial meningioma xenografts and was well-tolerated overall as treated mice did not show overt weight loss (Supplementary Fig. S13). Interestingly, upon temporary drug cessation, the combination-treated tumors took longer before they resumed growth, and tumor regression promptly occurred upon drug readministration. In contrast, single-agent INK128 showed transient tumor regression during the first week of retreatment before resuming growth, suggesting that the treated tumors may have developed partial resistance to INK128.

One limitation of this study is that only one NF2-SWN–associated meningioma model was used to evaluate the antitumor synergy of the brigatinib + INK128 combination; however, we also observed the antitumor synergy in the *NF2*-deficient Ben-Men-1 sporadic meningioma model. Given that each NF2-SWN patient may carry different *NF2* and other gene mutations, it would be imperative to establish additional NF2-SWN–related meningioma models as generation of a panel of these models would facilitate accurate identification of effective drugs or drug combinations by accounting for variations in treatment response. Ultimately, the effects of unique patient mutations can be studied, and therapeutic testing can be customized, signifying an advance in personalized healthcare. It should be noted that the immortalization method that we described can also be applied to other tumor types with limited growth capabilities, such as vestibular schwannomas.

Overall, we have described the AG-NF2-Men cell line as the first NF2-SWN–related meningioma cell line generated exclusively through telomerase immortalization. We also generated an orthotopic NF2-SWN–related meningioma model using luciferase-expressing AG-NF2-Men-Luc2 cells. In addition to the Ben-Men-1-LucB model (17), this model should serve as a valuable addition to identify novel drugs/drug combinations to treat NF2-SWN–related and *NF2*-deficient sporadic meningiomas. The enhanced antitumor effects of the brigatinib + INK128 combination that we observed indicate combining brigatinib with an mTOR inhibitor may be a promising treatment to improve the clinical care of NF2-SWN patients with meningiomas.

## Supplementary Material

Supplementary Data Supplementary Methods

Supplementary Figure S1 Figure S1. IHC analysis of AG-NF2-Men tumor sections for various NF2/merlin-regulated signaling molecules, cMYC, and CD163.

Supplementary Figure S2 Figure S2. Detection of the loss of a copy of chromosome 22 in primary and immortalized AG-NF2-Men cells.

Supplementary Figure S3 Figure S3. The AG-NF2-Men cell line exhibited a slow doubling time, consistent with the growth characteristics of a benign tumor.

Supplementary Figure S4 Figure S4. Analysis of the transcriptome of immortalized AG-NF2-Men cells reveals expression of several meningioma biomarkers and merlin-regulated RTKs.

Supplementary Figure S5 Figure S5. The AG-NF2-Men cell line expresses several RTKs, such IGF-1R, and responds to their cognate ligands.

Supplementary Figure S6 Figure S6. Serum-starvation of AG-NF2-Men cells blocked endogenous phosphorylation of EGFR, AKT, and ERK1/2.

Supplementary Figure S7 Figure S7. The brigatinib+INK128 combination promotes caspase-3/7 activation, indicative of apoptosis.

Supplementary Figure S8 Figure S8. The brigatinib+INK128 combination, but not the monotherapies, markedly downregulated several YAP target genes in treated AG-NF2-Men cells.

Supplementary Figure S9 Figure S9. Ingenuity Pathway analysis indicated that the activities of several upstream regulators important for meningioma growth were significantly altered in AG-NF2-Men cells treated with the brigatinib+INK128 combination.

Supplementary Figure S10 Figure S10. AG-NF2-Men-Luc2 cells did not grow when implanted subcutaneously but readily established intracranial xenografts when injected into the skull base of NSG mice.

Supplementary Figure S11 Figure S11. The brigatinib+INK128 combination more effectively shrank intracranial AG.

Supplementary Figure S12 Figure S12. Combining INK128 with brigatinib also enhanced anti-tumor effects in the sporadic NF2-deficient Ben-Men-1-LucB xenograft model.

Supplementary Figure S13 Figure S13. Treatment with brigatinib, INK128, or brigatinib+INK128 did not cause overt weight loss, compared with vehicle-treated mice.

Supplementary Data S1 Supplementary Data S1

Supplementary Data S2 Supplementary Data S2

## Data Availability

The RNA-seq data generated in this study are publicly available in Gene Expression Omnibus at GSE307286 (https://www.ncbi.nlm.nih.gov/geo/query/acc.cgi?acc=GSE307286). Analyzed data were provided as Supplementary Data files S1 and S2.
