# Supplementary material for: Combining Brigatinib with mTOR Inhibition to Effectively Treat NF2-SWN–Associated and Sporadic NF2-Deficient Meningiomas
Source: Cancer Res Commun. 2026 Jan 27;6(1):211–23. doi: 10.1158/2767-9764.CRC-25-0563 (PMC12835584; doi:10.1158/2767-9764.CRC-25-0563)
Supplement: Supplementary Data — Supplementary Methods [file crc-25-0563_supplementary_data_suppsm.pdf]

Supplementary Methods

Compounds. Brigatinib (CAS:1197953-54-0) and INK128 (CAS:1224844-38-5) were synthesized by Proactive Molecular Research with purities >98% (20,21). For *in vitro* studies, compounds were dissolved in DMSO as 10mM stocks. For mouse dosing, brigatinib was formulated as 5mg/mL solution in 90% polyethylene glycol-300 and 10% 1-methyl-2-pyrrolidinone, and INK128 as 0.075mg/mL solution in 5% 1-methyl-2-pyrrolidinone, 15% polyvinylpyrrolidone K30, and 80% water. For combination treatment, brigatinib and INK128 were prepared as 2X concentrates in each respective vehicle, and equal amounts of each drug solution were mixed to obtain the final concentration of 5mg/mL of brigatinib and 0.075mg/mL of INK128. All mice received 0.1mL of each formulated drug or drug combination per 10g of mouse weight everyday by oral gavage.

Preparation of primary meningioma cells and other cell cultures. Meningioma tumor tissues were acquired from patients with written informed consent under IRB-approved Human Subjects protocols for the accrual of tumor specimens from meningioma and schwannoma patients. Tumors were finely minced with scalpels, and the resulting fragments enzymatically dissociated with collagenase (0.6 U/ml) and dispase (0.125 U/ml) in Dulbecco's Modified Eagle's (DME) medium (all from ThermoFisher) at 37°C for 3 hours. Then, digested tumor cell suspension was triturated and pelleted by centrifugation at 300 x g. Cell pellets were resuspended in growth medium consisting of DME medium supplemented with 10% heat-inactivated fetal bovine serum (FBS; R&D Systems) and seeded for propagation in 10-cm cell culture dishes (25).

Ben-Men-1 (RRID:CVCL_1959) and its luciferase-expressing Ben-Men-1-LucB cells (CVCL_JK01) (Burns et al., 2013), AG-NF2-Men and its luciferase-expressing AG-NF2-Men-Luc2 cells, HeLa cells (RRID:CVCL_0030), normal meningeal cells (ScienCell Cat #1400), and primary meningioma cells generated from different patients as described above were grown in DMEM plus 10% FBS. All cell lines were authenticated by short tandem repeat genotyping and

tested to be mycoplasma-free.

Antibodies for immunohistochemistry (IHC) and Western blotting. For IHC,tumor sections were immunostained with antibodies against phospho-ErbB3[Y^{1289}] (p-ErbB3[Y^{1289}]; Cat# 4791, RRID:AB_2099709), p-Erk1/2[T^{202}/Y^{204}] (Cat# 4370, RRID:AB_2315112), p-AKT[S^{473}] (Cat# 4060, RRID:AB_2315049; all from Cell Signaling Technology), p-FAK[Y^{397}] (ThermoFisher Cat# 700255, RRID:AB_2532307), epithelial membrane antigen (EMA; Applied Biological Materials Cat# HY500076), CD163 (Biocare Medical Cat# CM353, RRID:AB_10583140), and c-MYC (ScyTek Laboratories Cat# RA0226) as previously described (Burns et al., 2013).

For Western blotting, the primary antibodies used include anti-merlin (Cat# 12888, RRID:AB_2650551), EGFR (Cat# 4267, RRID:AB_2246311), p-EGFR[Y^{1068}] (Cat# 3777, RRID:AB_2096270), p-ErbB3[Y^{1289}] (Cat# 4791, RRID:AB_2099709), IGF-1R (Cat# 9750, RRID:AB_10950969), p-IGF-1R[$Y^{1135/1136}$] (Cat# 3024, RRID:AB_331253), AKT (Cat# 2920, RRID:AB_1147620 and Cat# 4691, RRID:AB_915783), p-AKT[S^{473}] (Cat# 4060, RRID:AB_2315049), p-AKT[T^{308}] (Cat# 2965, RRID:AB_2255933), ERK1/2 (Cat# 4695, RRID:AB_390779), p-ERK1/2[T^{202}/Y^{204}] (Cat# 4370, RRID:AB_2315112), 4EBP1 (Cat# 9644, RRID:AB_2097841)), p-4EBP1[T^{65}] (Cat# 9451, RRID:AB_330947), p-4EBP1[$S^{37/46}$] (Cat# 2855, RRID:AB_560835), GAPDH (Cat# 5174, RRID:AB_10622025) (all from Cell Signaling Technology), desmoplakin 1/2 (GeneTex Cat# GTX41413, RRID:AB_11176103), α-tubulin (Cat# sc-32293, RRID:AB_628412)) and ErbB3 (Cat# sc-285, RRID:AB_2099723) (both from Santa Cruz Biotechnology).

Telomerase-expressing retrovirus, immortalization, telomerase assay, and fluorescence in situ hybridization (FISH). Human telomerase reverse transcriptase (hTERT)-expressing retroviruses were produced by co-transfecting the hTERT-expressing retroviral plasmid pLNCX2-hTERT, which also contains a neomycin-resistance gene and can confer G418-resistance when expressed in mammalian cells, and pVSV-G into GP2-293 packaging

cells (Takara Bio) by the calcium phosphate technique. After 24 and 48h, the supernatant containing retroviral particles was collected and filtered through a 0.45-μm filter (Corning). For immortalization, primary meningioma cells were infected with 1 mL of supernatant containing hTERT-expressing retroviruses in the presence of 8 mg/mL polybrene at 37°C overnight (Burns and Chang, 2016). Infected cells were passaged in medium without G418 selection until immortalized cells emerged. The telomerase-immortalized cells were subcloned in G418-containing medium (500μg/ml).

The telomerase activities in primary and immortalized AG-NF2-Men cells were measured using the Telomerase Activity Quantification qPCR Assay Kit, which detects telomeric repeats added to the 3' end of a telomerase-specific substrate (ScienCell #8928) according to manufacturer's instructions. Real-time quantitative PCR amplification of telomeric repeats was performed using a Bio-Rad CFX96 Touch thermocycler (RRID:SCR_018064). The telomerase activity of immortalized AG-NF2-Men cells was estimated relative to primary meningioma cells by the ΔC_t method. To confirm amplification of a specific product, melt curve analysis was performed from 65 to 95°C in 0.5°C increments, and the ~75-bp PCR product electrophoresed in a 3.5% agarose gel in Tris-acetate/EDTA buffer.

FISH analysis was performed using the Vysis EWSR1 (22q12) Dual Color Break Apart Rearrangement FISH Probe Kit (Abbott Molecular) on nuclei from the primary and immortalized meningioma cells according to the manufacturer's instructions. The probe kit contains two DNA probes from the chromosome 22q12 region in which the *NF2* gene is located. The first is a 497-kb probe labeled in Spectrum Orange and flanking the 5' side of the EWSR1 gene. The second is a 1,100-kb DNA labeled in Spectrum Green and extends from the 3' end of the EWSR1 gene to the sequence beyond the *NF2* gene. In a cell with two intact copies of chromosome 22, a two-red:green fusion signal pattern is observed, while only one red:green fusion signal is seen in the cell with only one chromosome 22.

Resazurin assays, drug combination matrix arrays, and cell counting. AG-NF2-Men

cells were seeded in 96-well plates (Sarstedt) at 4,000 cells/well and allowed to adhere overnight. Brigatinib and INK128 were added to cells as 9-point, 2-fold serial dilutions in growth medium with 4-12 replicate wells per treatment dose. Cell proliferation was assessed after 3 days by adding resazurin and measuring metabolic conversion to fluorescent resorufin (excitation wavelength = 544nm; emission wavelength = 590nm) on a SpectraMax M2e plate reader (Molecular Devices). Percent viability was calculated by averaging fluorescence values of drug-treated, replicate wells and then normalizing to the DMSO control set as 100% (Burns et al., 2013). GraphPad Prism v10 (RRID:SCR_002798) was used for plotting the dose-response curves using nonlinear regression analysis for curve fitting, and the mean absolute IC_{50} (50% inhibitory concentration) values estimated from the curves. For drug combinations, AG-NF2-Men (4,000 cells/well) and Ben-Men-1 cells (2,000 cells/well) were seeded as 8x8 matrices in 96-well plates. The following day, brigatinib and INK128 were arrayed in combination using a 7-point, 2-fold dilution series. Cell proliferation of Ben-Men-1 cells was measured by resazurin assay after 3-day treatment. Due to slower population doubling times, AG-NF2-Men cell proliferation was assessed after being treated for 7 days or longer. SynergyFinder v3.0 was used to calculate synergy scores according to the Loewe additivity model (Ianevski et al., 2022; RRID:SCR_026127; https://synergyfinder.fimm.fi/). In addition, the growth-inhibitory synergy of the brigatinib+INK128 combination in AG-NF2-Men cells was also assessed by cell counting. Cells were seeded at 2,000 cells/cm^2 in 6-well plates (Sarstedt) and treated the following day with 1x IC_{50} of brigatinib, INK128, or brigatinib+INK128, or DMSO as controls. Duplicate wells of treated cells were counted by hemocytometer every 3-4 days, and media and compounds were refreshed on unharvested wells.

Incucyte live cell imaging of caspase-3/7 cleavage for apoptosis. AG-NF2-Men cells were seeded at 4,000 cells/well in 96-well plates and treated in triplicate wells with 1x IC50 of brigatinib, INK128, or brigatinib+INK128 in the presence of Incucyte Caspase-3/7 Green Apoptosis Assay Reagent (#4440, Sartorius). AG-NF2-Men cells treated with 300nM

staurosporine were used as positive controls for caspase-3/7 activation, and untreated AG-NF2-Men cells without supplemented Caspase-3/7 Green reagent were assessed as negative controls. Plates were scanned on an Incucyte® live-cell imaging system (RRID:SCR_023147) over 9 days with 5 micrographs acquired per well at 4-hour intervals. On days 2 and 6, media with DMSO, brigatinib, INK128, and Caspase-3/7 Green reagent were refreshed. Green-fluorescence staining counts were used to quantify apoptosis from each micrograph, and data were plotted in GraphPad Prism as the mean ± SEM.

Western blots and immunofluorescence staining. Actively-growing AG-NF2-Men, Ben-Men-1, HeLa, and primary normal meningeal cells (ScienCell), as well as primary meningioma cells prepared from four NF2-SWN tumors were lysed in cold Triton X-100 lysis buffer (Chang et al., 2021). Equal amounts of protein lysates were resolved on SDS-polyacrylamide gels, electroblotted onto Immobilon-FL membranes (MilliporeSigma), and then probed with various indicated antibodies. Primary antibody-bound proteins were detected using IRDye-conjugated secondary antibodies, followed by scanning on an Odyssey CLx Imaging System (LI-COR; RRID:SCR_014579) using the appropriate fluorescent channel at a resolution of 84 μm. Also, AG-NF2-Men cells were starved overnight in serum-free DMEM. The next day, starved cells were stimulated for 10 minutes with 50 ng/mL of EGF, heregulin (Hrg), or IGF-1 and then lysed for Western blotting. To investigate the effects of drug treatment, subconfluent AG-NF2-Men cells were treated for 1 and 3 days with 1x IC_{50} of brigatinib, INK128, or brigatinib+INK128 and then lysed as described above. Also, serum-starved cells were pretreated for 2 hours with 1x IC_{50} of brigatinib, INK128, or brigatinib+INK128 and then stimulated for 5 minutes with 50 ng/mL of EGF, heregulin (Hrg), or IGF-1, followed by cell lysis and Western blotting.

For immunofluorescence staining, AG-NF2-Men cells were plated at $2x10^4$ cells/cm^2 on sterile glass coverslips. The following day attached cells were washed in phosphate-buffered saline (PBS) and fixed at room temperature (RT) for 30 min in 4% paraformaldehyde. Fixed cells were blocked with 10% bovine serum albumin in PBS, permeabilized for 15 min at RT in

0.1% Triton X-100, and incubated with an anti-vimentin antibody (Abcam Cat# ab16700, RRID:AB_443435) for 2h at RT and then an Alexa Fluor 488-conjugated goat anti-rabbit secondary antibody (Molecular Probes Cat# A11029, RRID:AB_2534088) for 1h at RT. Following washing with PBS four times, nuclei of stained cells were counterstained for 10 min at RT with 0.2 µg/mL of 4′,6-diamidino-2-phenylindole (DAPI) in PBS. Immunofluorescent images were captured using a Leica DM IRB inverted UV microscope attached to a SPOT digital camera imaging system (RRID:SCR_016613).

RNA-sequencing (RNA-seq) analysis. AG-NF2-Men cells were plated at 15,000 cells/cm2 in 10-cm dishes and treated in triplicate with 1x IC50 of brigatinib, INK128, brigatinib+INK128, or an equivalent amount of DMSO (0.01%) in fresh DMEM plus 10% FBS. After 24-h incubation, cells were scraped off dishes, washed twice with cold PBS, and flash-frozen at -80°C until shipment on dry ice to MedGenome. Total RNA was isolated from drug-treated cells using the Maxwell RSC simplyRNA Cells kit (Promega). Libraries were prepared with the Illumina TruSeq stranded mRNA sample preparation kit, and 100-bp paired-end reads were sequenced on an Illumina NovaSeq6000 sequencing system (RRID:SCR_016387). After excluding non-poly(A)-tailed RNA sequences with Bowtie2 (v.2.5.1) (Langmead and Salzberg, 2012; RRID:SCR_005476), reads were mapped by STAR v2.7.3a (Dobin et al., 2013; RRID:SCR_004463) to the GRCh37/hg19 human reference genome. Raw reads estimated by HTSeq v0.11.2 (Anders et al., 2015; RRID:SCR_005514) were normalized with DESeq2 (Love et al., 2014; RRID:SCR_015687). Gene expression levels in FPKM (fragments transcript kilobase per million) were determined in Cufflinks v2.2.1 (Trapnell et al., 2012; RRID:SCR_014597). Differentially-expressed genes (DEGs) were calculated by DESeq2. Significant DEGs were defined as transcripts with absolute log2 fold-changes (log2 FC) ≥ 1 and adjusted P-values (Padj) ≤ 0.01. Principal component analysis was generated by AltAnalyze using the significantly differentially regulated gene set in each drug-treated group (Emig et al., 2010; RRID:SCR_002951). Overlapping DEG were graphed as proportional Venn diagrams

using DeepVenn (https://deepvenn.com). Volcano plots for DEGs were constructed using the Galaxy Project bioinformatics website (RRID:SCR_006281; https://usegalaxy.org). Predicted upstream signaling regulators activated or inhibited in drug-treated AG-NF2-Men cells were ascertained by the Ingenuity Pathway Analysis (IPA) Upstream Analysis module (Qiagen; RRID:SCR_008653), following Core Analysis of the transcriptomic dataset with the cutoffs: Padj<0.01, absolute log2 FC >1, mean normalized read counts > 5.

RNA-seq data were deposited into the Gene Expression Omnibus database under accession number GSE307286 and are available at the following URL: https://www.ncbi.nlm.nih.gov/geo/query/acc.cgi?acc=GSE307286. Analyzed data were provided as Supplementary data files S1 and S2.

Generation of skull-base meningioma xenograft models. Immortalized AG-NF2-Men cells were infected with luciferase-expressing lentiviruses as previously described (17). Antibiotic-resistant colonies were selected and assessed for luciferase activity using the One-GLO™ Luciferase Assay System (Promega). The clone AG-NF2-Men-Luc2, expressing the highest luciferase activity, was chosen for animal implantation. All animal procedures were performed according to the protocol approved by the Institutional Animal Care and Use Committee of Nationwide Children's Hospital. AG-NF2-Men-Luc2 or Ben-Men-1-LucB cells ($5x10^5$ cells/mouse) were stereotactically into 8-to-12-week-old NSG mice (*NOD.Cg-Prkdc*^{scid} *Il2rg*^{tmlWjl}*/SzJ*; The Jackson Laboratory RRID:IMSR_JAX:005557) as previously described (Burns et al., 2013), and injected mice were monitored by weekly bioluminescence imaging (BLI) using a Spectrum *In Vivo* Imaging System (Revvity; RRID:SCR_018621). We previously showed that tumor-emitted luminescence correlates with tumor volume as measured by MRI. Mice with established tumors, defined as increased in BL signals over at least two consecutive timepoints, were randomized into different treatment groups and received vehicle, 50mg/kg of brigatinib, 0.75mg/kg of INK128, or their combination (n=10/group) every day by oral gavage. The effects of treatment were monitored by BLI. After 8-week treatment, we stopped treating a

cage of tumor-bearing mice that had received INK128 or the brigatinib+INK128 combination and monitored for possible tumor regrowth for six more weeks. Then, we retreated these mice to determine whether regrown tumors were still drug sensitive.

References

Burns SS, Akhmametyeva EM, Oblinger JL, Bush ML, Huang J, Senner V, Chen C-S, Jacob A, Welling DB, Chang L-S. Histone deacetylase inhibitor AR-42 differentially affects cell-cycle transit in meningeal and meningioma cells, potently inhibiting *NF2*-deficient meningioma growth. Cancer Res. 2013;73:792-804.

Burns SS, Chang L-S. Generation of noninvasive, quantifiable, orthotopic animal models for NF2-associated schwannoma and meningioma. Methods Mol Biol. 2016;1427:59-72.

Chang LS, Oblinger JL, Smith AE, Ferrer M, Angus SP, Hawley E, Petrilli AM, Beauchamp RL, Riecken LB, Erdin S, Poi M, Huang J, Bessler WK, Zhang X, Guha R, Thomas C, Burns SS, Gilbert TSK, Jiang L, Li X, Lu Q, Yuan J, He Y, Dixon SAH, Masters A, Jones DR, Yates CW, Haggarty SJ, La Rosa S, Welling DB, Stemmer-Rachamimov AO, Plotkin SR, Gusella JF, Guinney J, Morrison H, Ramesh V, Fernandez-Valle C, Johnson GL, Blakeley JO, Clapp DW; Synodos for NF2 Consortium. Brigatinib causes tumor shrinkage in both *NF2*-deficient meningioma and schwannoma through inhibition of multiple tyrosine kinases but not ALK. PLoS One. 2021;16:e0252048.

Ianevski A, Giri AK, Aittokallio T. SynergyFinder 3.0: an interactive analysis and consensus interpretation of multi-drug synergies across multiple samples. Nucleic Acids Res. 2022;50(W1):W739-W743.

Van der Eb AJ, Graham FL. Assay of transforming activity of tumor virus DNA. Methods Enzymol. 1980;65:826-839.
