# Supplementary material for: Combining Brigatinib with mTOR Inhibition to Effectively Treat NF2-SWN–Associated and Sporadic NF2-Deficient Meningiomas
Source: Cancer Res Commun. 2026 Jan 27;6(1):211–23. doi: 10.1158/2767-9764.CRC-25-0563 (PMC12835584; doi:10.1158/2767-9764.CRC-25-0563)

Supplementary Figure S1. IHC analysis of AG-NF2-Men tumor sections for various *NF2*/merlin-regulated signaling molecules, cMYC, and CD163. Tissue sections of the AG-NF2-Men tumor (A, D, G, J, M, and P), a normal human sciatic nerve (B, E, H, K, N, Q, and R), and normal mouse brain (C, F, I, L, O, and R) were immuno-stained for p-ErbB3(Y1289) (A-C), p-FAK(Y397) (D-F), p-AKT(S473) (G-I), p-ERK1/2(T202/Y204) (J-L), cMYC (M-O), and CD163 (P-R) according to Methods. The meningeal cell layer covering the mouse brain (B) was denoted with an arrow. S, Skull.

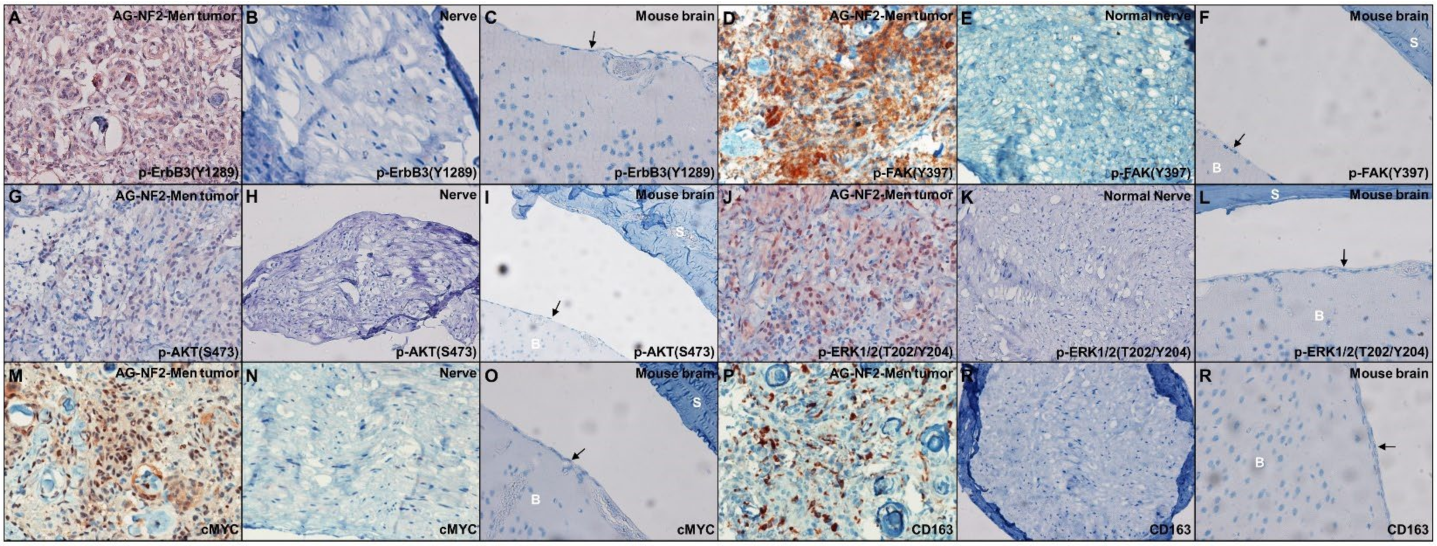

Supplement: Supplementary Figure S1 — Figure S1. IHC analysis of AG-NF2-Men tumor sections for various NF2/merlin-regulated signaling molecules, cMYC, and CD163. [file crc-25-0563_supplementary_figure_s1_suppsf1.pdf]
