# Supplementary material for: Combining Brigatinib with mTOR Inhibition to Effectively Treat NF2-SWN–Associated and Sporadic NF2-Deficient Meningiomas
Source: Cancer Res Commun. 2026 Jan 27;6(1):211–23. doi: 10.1158/2767-9764.CRC-25-0563 (PMC12835584; doi:10.1158/2767-9764.CRC-25-0563)

Supplementary Figure S2. Detection of the loss of a copy of chromosome 22 in primary and immortalized AG-NF2-Men cells. FISH analysis was performed on the primary AG-NF2-Men cells prepared from an NF2-SWN patient's meningioma (A,B) and the telomerase-immortalized AG-NF2-Men cell line (C) using the Vysis LSI EWSR1 (22q12) Dual Color Break Apart Rearrangement FISH Probe Kit. The majority of primary AG-NF2-Men tumor cells showed one colocalized red:green signal (A,B), indicating the presence of only one copy of chromosome 22. As primary tumor cell cultures are often mixed with normal stromal cells which have two copies of chromosome 22, some of the cells in the primary AG-NF2-Men tumor culture exhibited two red:green fusion signals (arrows in panel B). Importantly, only one co-localized red:green signal was detected in immortalized AG-NF2-Men cells, as seen in the parental tumor cells.

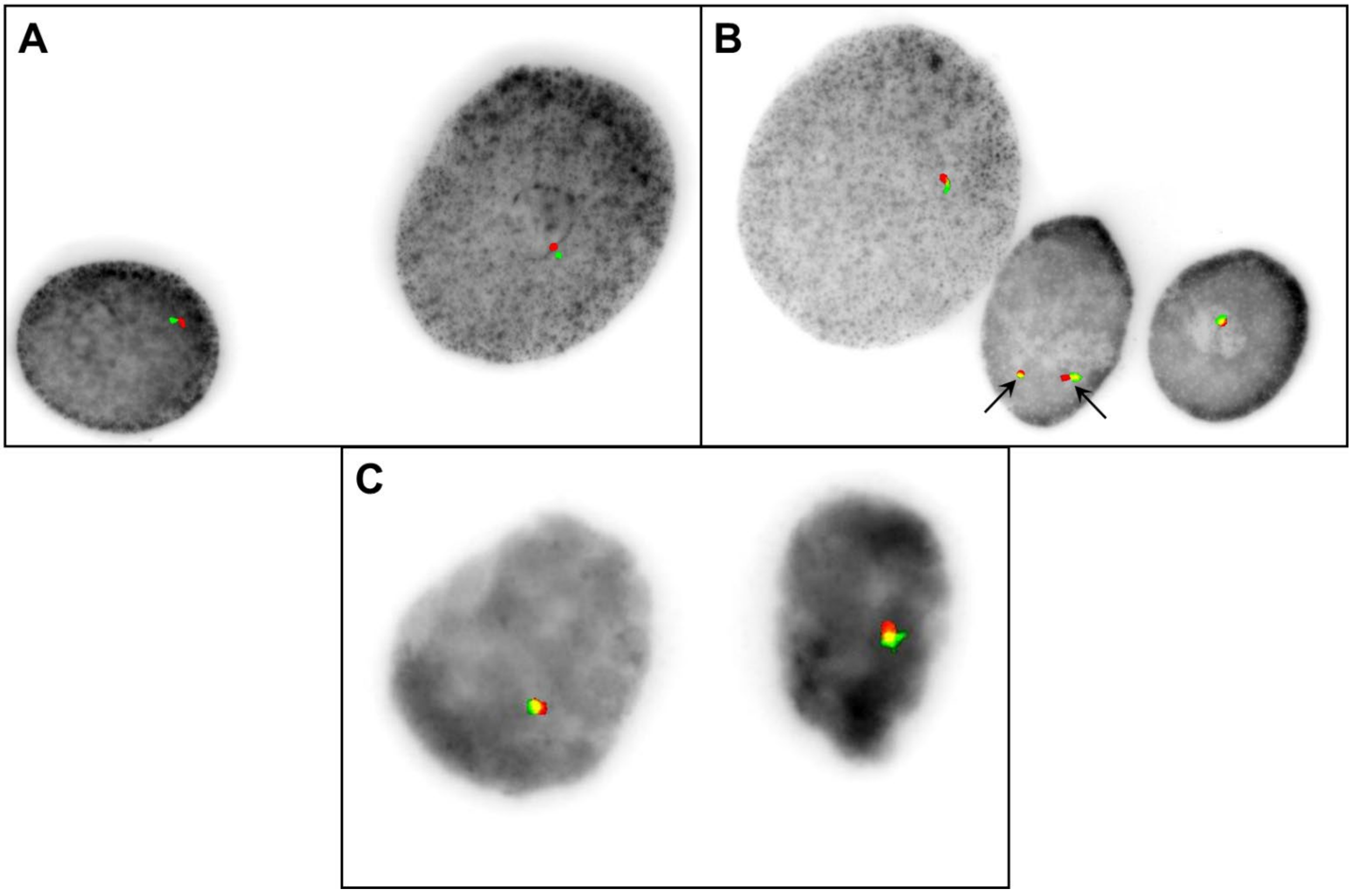

Supplement: Supplementary Figure S2 — Figure S2. Detection of the loss of a copy of chromosome 22 in primary and immortalized AG-NF2-Men cells. [file crc-25-0563_supplementary_figure_s2_suppsf2.pdf]
