# Supplementary material for: Combining Brigatinib with mTOR Inhibition to Effectively Treat NF2-SWN–Associated and Sporadic NF2-Deficient Meningiomas
Source: Cancer Res Commun. 2026 Jan 27;6(1):211–23. doi: 10.1158/2767-9764.CRC-25-0563 (PMC12835584; doi:10.1158/2767-9764.CRC-25-0563)

Supplementary Figure S3. The AG-NF2-Men cell line exhibited a slow doubling time, consistent with the growth characteristics of a benign tumor. Immortalized AG-NF2-Men cells seeded in 6-well plates were trypsinized and counted on a hemocytometer every 2-3 days. The graph shows the mean and standard deviation of the cell numbers for each indicated timepoint from two independent experiments. The calculated population doubling time with 95% confidence interval (CI) for the AG-NF2-Men cell line is 2.5 to 2.6 days. Clear squares show the cell counts from individual wells for each experiment.

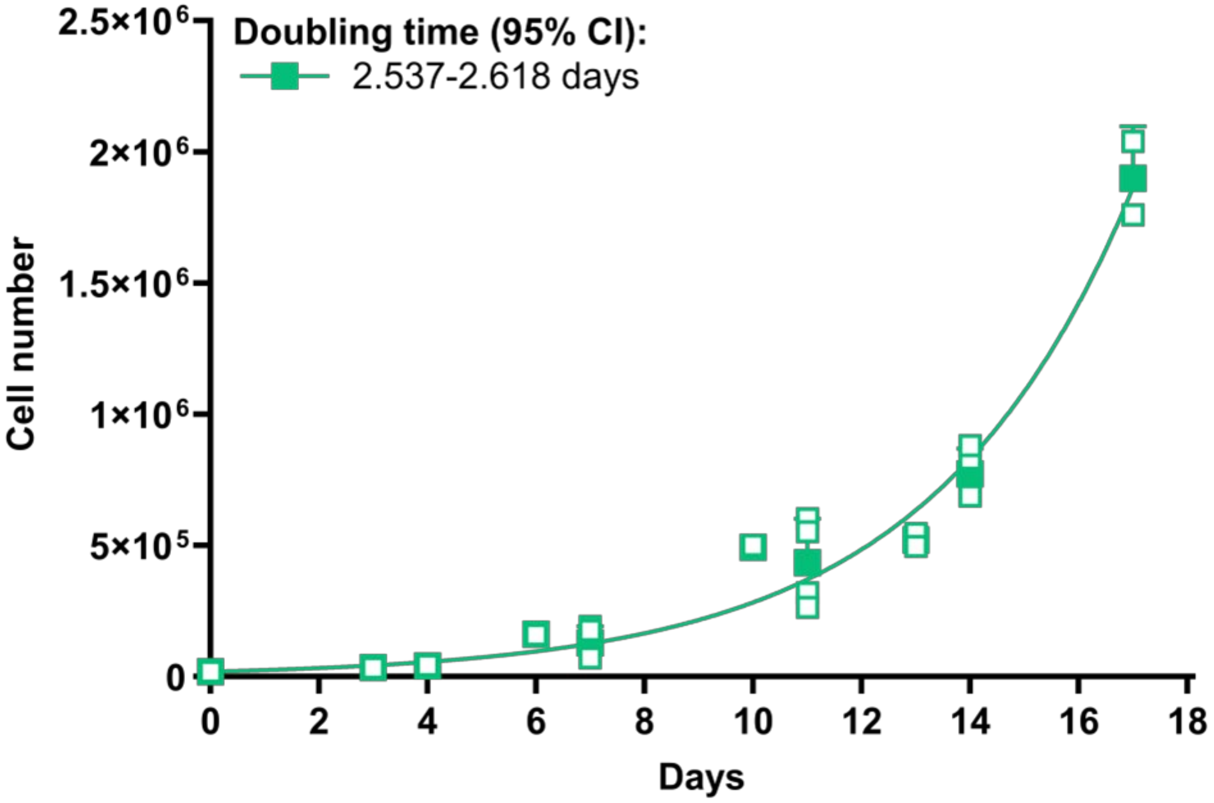

Supplement: Supplementary Figure S3 — Figure S3. The AG-NF2-Men cell line exhibited a slow doubling time, consistent with the growth characteristics of a benign tumor. [file crc-25-0563_supplementary_figure_s3_suppsf3.pdf]
