# Supplementary material for: Combining Brigatinib with mTOR Inhibition to Effectively Treat NF2-SWN–Associated and Sporadic NF2-Deficient Meningiomas
Source: Cancer Res Commun. 2026 Jan 27;6(1):211–23. doi: 10.1158/2767-9764.CRC-25-0563 (PMC12835584; doi:10.1158/2767-9764.CRC-25-0563)
Supplement: Supplementary Figure S4 — Figure S4. Analysis of the transcriptome of immortalized AG-NF2-Men cells reveals expression of several meningioma biomarkers and merlin-regulated RTKs. [file crc-25-0563_supplementary_figure_s4_suppsf4.pdf]

Supplementary Figure S4. Analysis of the transcriptome of immortalized AG-NF2-Men cells reveals expression of several meningioma biomarkers and merlin-regulated RTKs. RNA-sequencing was performed according to Methods. Shown are the average normalized transcript read counts (expressed as FPKM) and individual normalized read counts from three biological replicates of AG-NF2-Men cells for mRNAs encoding (A) various meningioma markers, including desmosomal components and (B) growth factors and their cognate RTKs

A

Ensembl ID	Gene Symbol	Average Normalized Read Counts	Replicate 1	Replicate 2	Replicate 3
ENSG00000096696	*DSP*	42609.15	42678.22	42165.80	42983.42
ENSG00000134760	*DSG1*	0.00	0.00	0.00	0.00
ENSG00000046604	*DSG2*	751.27	592.00	945.67	716.15
ENSG00000175065	*DSG4*	0.00	0.00	0.00	0.00
ENSG00000134757	*DSG3*	0.32	0.00	0.95	0.00
ENSG00000134765	*DSC1*	1.05	0.00	0.00	3.14
ENSG00000134755	*DSC2*	5.95	1.90	7.60	8.36
ENSG00000134762	*DSC3*	171.95	167.92	198.44	149.50
ENSG00000081277	*PKP1*	0.98	0.95	0.95	1.05
ENSG00000057294	*PKP2*	1696.98	1805.42	1803.04	1482.48
ENSG00000184363	*PKP3*	2.91	5.69	0.95	2.09
ENSG00000144283	*PKP4*	12471.01	12008.91	12818.74	12585.36
ENSG00000185499	*MUC1 (EMA)*	1334.56	1415.49	1037.77	1550.43
ENSG00000180616	*SSTR2*	0.00	0.00	0.00	0.00
ENSG00000026025	*VIM*	388770.75	416657.48	377355.88	372298.90

B

Ensembl ID	Gene Symbol	Average Normalized Read Counts	Replicate 1	Replicate 2	Replicate 3
ENSG00000138798	*EGF*	10.44	16.13	15.19	0.00
ENSG00000146648	*EGFR*	35554.53	31570.60	40530.82	34562.17
ENSG00000157168	*NRG1*	371.43	356.72	396.88	360.69
ENSG00000158458	*NRG2*	2.85	5.69	2.85	0.00
ENSG00000141736	*ERBB2*	1665.93	2101.42	791.85	2104.53
ENSG00000065361	*ERBB3*	349.14	342.49	276.29	428.64
ENSG00000178568	*ERBB4*	14.81	20.87	15.19	8.36
ENSG00000017427	*IGF1*	1.90	0.00	5.70	0.00
ENSG00000140443	*IGF1R*	7781.55	7861.10	7370.71	8112.84
ENSG00000112715	*VEGFA*	1020.33	1042.64	728.24	1290.11
ENSG00000173511	*VEGFB*	3785.90	4635.45	2275.87	4446.38
ENSG00000150630	*VEGFC*	5900.05	5558.56	6498.15	5643.44
ENSG00000165197	*FIGF (VEGFD)*	3.10	0.95	0.00	8.36
ENSG00000102755	*FLT1 (VEGFR1)*	2.85	5.69	2.85	0.00
ENSG00000128052	*KDR (VEGFR2)*	285.51	268.49	274.40	313.64
ENSG00000171094	ALK	17.13	16.13	2.85	32.41
