# Supplementary material for: Combining Brigatinib with mTOR Inhibition to Effectively Treat NF2-SWN–Associated and Sporadic NF2-Deficient Meningiomas
Source: Cancer Res Commun. 2026 Jan 27;6(1):211–23. doi: 10.1158/2767-9764.CRC-25-0563 (PMC12835584; doi:10.1158/2767-9764.CRC-25-0563)

Supplementary Figure S5. The AG-NF2-Men cell line expresses several RTKs, such as EGFR, ErbB3, and IGF-1R, and responds to their cognate ligands. AG-NF2-Men cells were serum-starved overnight and then stimulated for 10 minutes with EGF, Hrg, or IGF-1. Lysates from these cells were analyzed by Western blotting for phosphorylation of the indicated growth factor receptors and their downstream targets AKT and ERK1/2. GAPDH served as a loading control. kD, kilodalton of molecular weight.

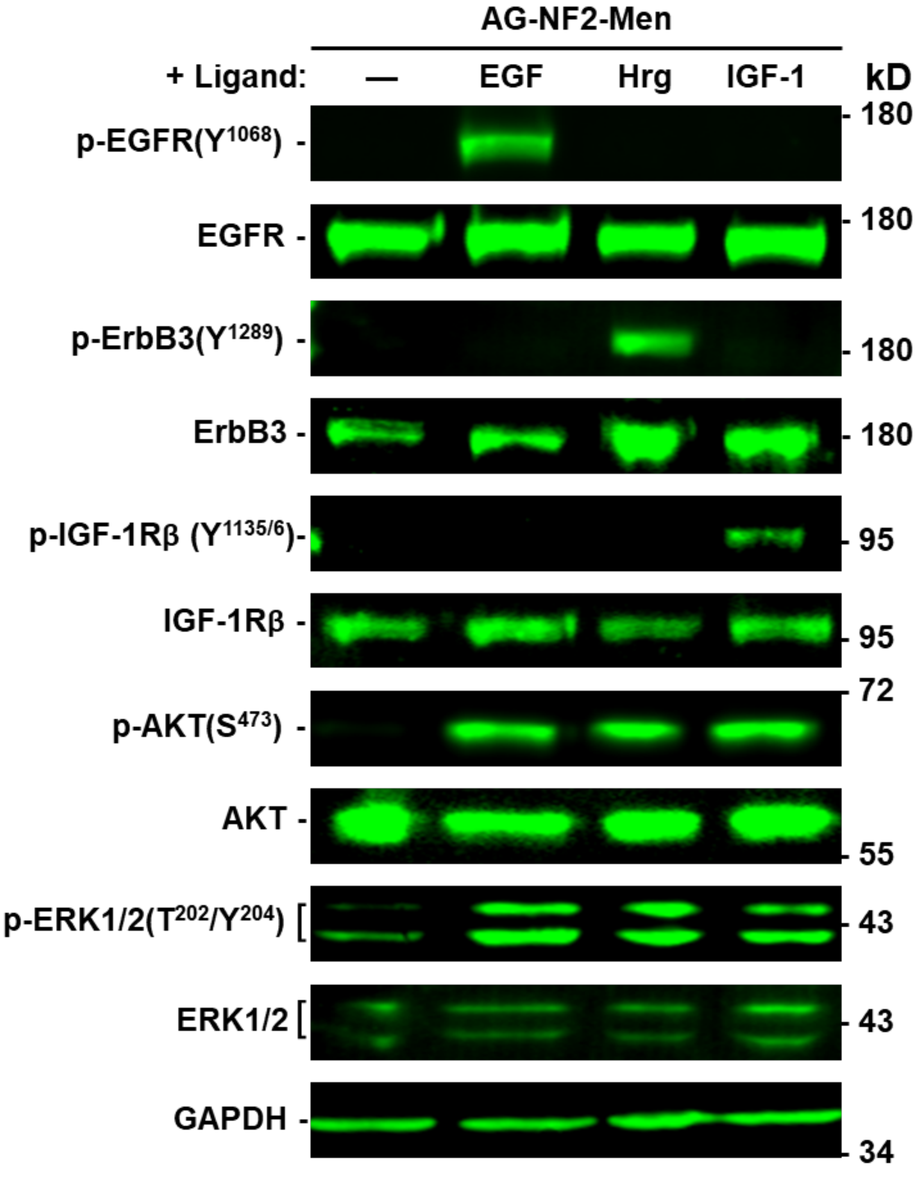

Supplement: Supplementary Figure S5 — Figure S5. The AG-NF2-Men cell line expresses several RTKs, such IGF-1R, and responds to their cognate ligands. [file crc-25-0563_supplementary_figure_s5_suppsf5.pdf]
