# Supplementary material for: Combining Brigatinib with mTOR Inhibition to Effectively Treat NF2-SWN–Associated and Sporadic NF2-Deficient Meningiomas
Source: Cancer Res Commun. 2026 Jan 27;6(1):211–23. doi: 10.1158/2767-9764.CRC-25-0563 (PMC12835584; doi:10.1158/2767-9764.CRC-25-0563)

Supplementary Figure S6. Serum-starvation of AG-NF2-Men cells blocked endogenous phosphorylation of EGFR, AKT, and ERK1/2. AG-NF2-Men cells were serum-starved for 24h to induce quiescence and then treated with brigatinib, INK128, brigatinib+INK128, or DMSO vehicle in serum-free medium for 2h. Treated cells were harvested, and lysates were analyzed for phosphorylation of the indicated RTKs and downstream AKT/PRAS40 and ERK1/2. Note that little or no p-EGFR, p-AKT, p-PRAS40, and p-ERK1/2 were detected in quiescent AG-NF2-Men cells with or without brigatinib treatment. Intriguingly, a modest increase in the p-ERK1/2 level was observed in growth-arrested AG-NF2-Men cells treated with INK128 or its combination with brigatinib, likely due to feedback signaling from mTOR inhibition. kD, kilodalton of molecular weight.

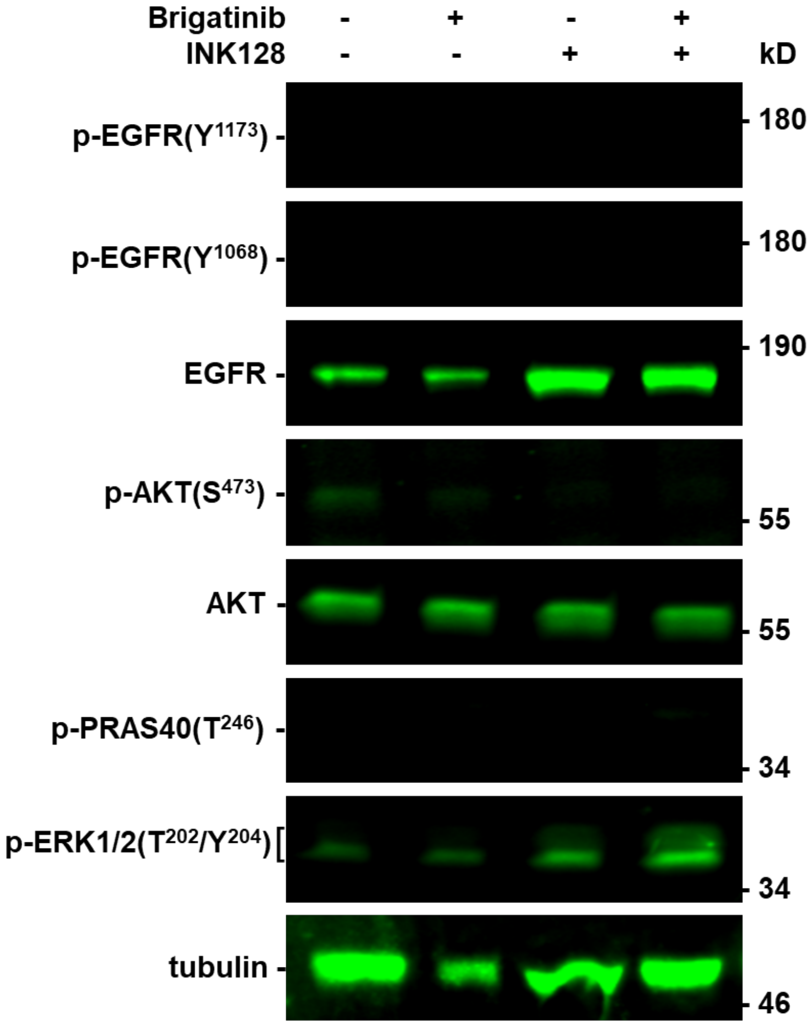

Supplement: Supplementary Figure S6 — Figure S6. Serum-starvation of AG-NF2-Men cells blocked endogenous phosphorylation of EGFR, AKT, and ERK1/2. [file crc-25-0563_supplementary_figure_s6_suppsf6.pdf]
