# Supplementary material for: Combining Brigatinib with mTOR Inhibition to Effectively Treat NF2-SWN–Associated and Sporadic NF2-Deficient Meningiomas
Source: Cancer Res Commun. 2026 Jan 27;6(1):211–23. doi: 10.1158/2767-9764.CRC-25-0563 (PMC12835584; doi:10.1158/2767-9764.CRC-25-0563)
Supplement: Supplementary Figure S7 — Figure S7. The brigatinib+INK128 combination promotes caspase-3/7 activation, indicative of apoptosis. [file crc-25-0563_supplementary_figure_s7_suppsf7.pdf]

Supplementary Figure S7. The brigatinib+INK128 combination promotes caspase-3/7 activation, indicative of apoptosis. (A) AG-NF2-Men meningioma cells were plated in a 96-well plate and treated with 1x IC_{50} of brigatinib, INK128, or brigatinib+INK128 or the equivalent DMSO vehicle (n=3 wells per treatment). An additional well of meningioma cells was treated with 300 nM staurosporine as a positive control for caspase-3/7 activation. Two more wells of untreated AG-NF2-Men cells without supplemented Caspase-3/7 Green reagent (“No Fluorophore) were included as negative controls for imaging in the green fluorescence channel. Treated cells were imaged on Incucyte® live-cell imaging system over 9 days, with 5 photographed regions per well. Media with DMSO, brigatinib, INK128, and Caspase-3/7 Green reagent were refreshed on days 2 and 6. Associated drops in cleaved caspase-positive labelling observed at the subsequent imaging scans were likely due to the detachment and removal of dead cells after media refresh. The graph depicts the 9-day treatment course and shows that the brigatinib and INK128 monotherapies have little caspase activation similar to the DMSO controls, while the brigatinib+INK128 combination elicits an increase in cleaved caspase positive labelling starting after 2-days of drug treatment. The graphs show the means ± SEMs of 15 photographed regions in drug- or DMSO-treated wells and n=5 regions for the staurosporine-treated well. (B) Representative phase contrast micrographs of cells after ~9 days of treatment with overlaid green fluorescence indicating cells with cleaved caspase-3/7.

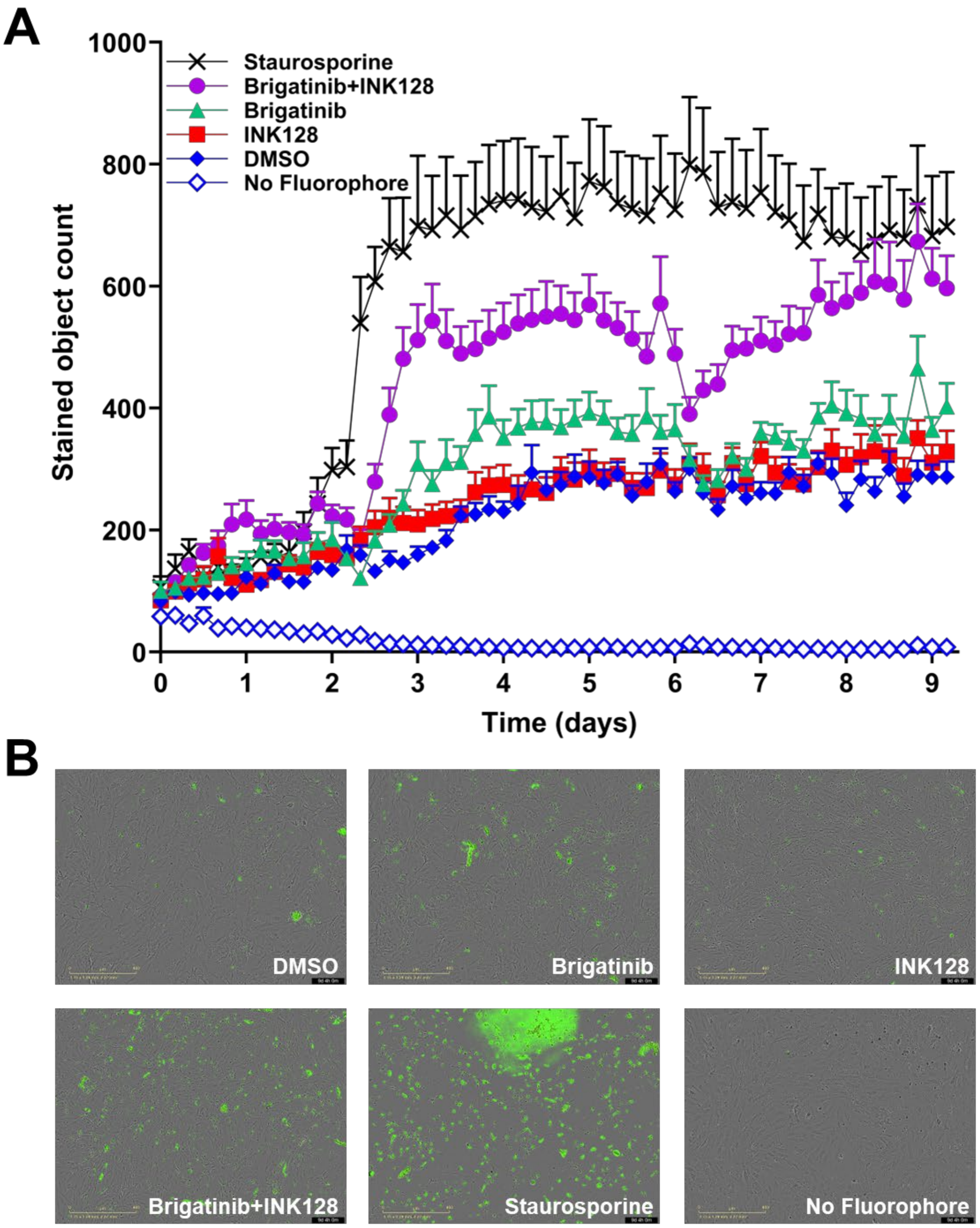
A
Staurosporine
Brigatinib+INK128
Brigatinib
INK128
DMSO
No Fluorophore
Stained object count
Time (days)
1000
800
600
400
200
0
0
1
2
3
4
5
6
7
8
9
B
DMSO
Brigatinib
INK128
Brigatinib+INK128
Staurosporine
No Fluorophore
