# Supplementary material for: Combining Brigatinib with mTOR Inhibition to Effectively Treat NF2-SWN–Associated and Sporadic NF2-Deficient Meningiomas
Source: Cancer Res Commun. 2026 Jan 27;6(1):211–23. doi: 10.1158/2767-9764.CRC-25-0563 (PMC12835584; doi:10.1158/2767-9764.CRC-25-0563)
Supplement: Supplementary Figure S8 — Figure S8. The brigatinib+INK128 combination, but not the monotherapies, markedly downregulated several YAP target genes in treated AG-NF2-Men cells. [file crc-25-0563_supplementary_figure_s8_suppsf8.pdf]

Supplementary Figure S8. The brigatinib+INK128 combination, but not the monotherapies, markedly downregulated several YAP target genes in treated AG-NF2-Men cells. Selected genes that are transcriptionally upregulated by YAP are listed. Shown are the average normalized read counts for the DMSO control, the brigatinib and INK128 monotherapies, and their combination. The ranking of the DEGs found in the combination-treated AG-NF2-Men cells was based on the nominal P-value, with lower numbers representing higher significance values. The log2 FC and Padj for the single and combination drug therapies were calculated relative to the DMSO control. The shading on log2 FC values indicates increased expression (red) or decreased expression (blue).

		Average Normalized Read Counts				Brigatinib + INK128 DEG Rank	Brigatinib		INK128		Brigatinib+INK128	
Ensembl ID	Gene Symbol	DMSO	Brigatinib	INK128	Brigatinib + INK128		log2 FC	Padj	log2 FC	Padj	log2 FC	Padj
ENSG00000148677	*ANKRD1*	204916	171904	243807	871	1	**-0.24**	6.42E-01	**0.23**	5.27E-01	**-7.77**	3.35E-199
ENSG00000128510	*CPA4*	9030	11178	8884	133	7	**0.26**	5.85E-01	**-0.03**	9.46E-01	**-6.00**	4.84E-131
ENSG00000118523	*CTGF*	93020	105809	153399	1698	9	**0.16**	7.91E-01	**0.66**	1.03E-02	**-5.71**	4.51E-125
ENSG00000142871	*CYR61*	59988	53189	46740	1274	29	**-0.17**	8.15E-01	**-0.33**	4.09E-01	**-5.48**	1.13E-95
ENSG00000114019	*AMOTL2*	7518	7640	8948	965	262	**0.001**	9.99E-01	**0.22**	5.47E-01	**-2.93**	3.49E-38
ENSG00000167601	*AXL*	70389	55016	70737	13588	263	**-0.35**	3.39E-01	**-0.0002**	9.99E-01	**-2.36**	5.35E-38
ENSG00000138772	*ANXA3*	14608	18219	18624	1814	307	**0.28**	5.57E-01	**0.32**	3.10E-01	**-2.98**	2.90E-35
ENSG00000129474	*AJUBA*	7949	9104	7370	2196	701	**0.16**	7.43E-01	**-0.11**	7.43E-01	**-1.84**	6.17E-22
ENSG00000135046	*ANXA1*	80631	87575	75488	38291	2948	**0.10**	8.77E-01	**-0.10**	7.89E-01	**-1.07**	2.71E-07
ENSG00000164442	*CITED2*	16733	27369	21314	11711		**0.62**	8.93E-02	**0.31**	3.95E-01	**-0.51**	6.40E-02
