# Supplementary material for: Combining Brigatinib with mTOR Inhibition to Effectively Treat NF2-SWN–Associated and Sporadic NF2-Deficient Meningiomas
Source: Cancer Res Commun. 2026 Jan 27;6(1):211–23. doi: 10.1158/2767-9764.CRC-25-0563 (PMC12835584; doi:10.1158/2767-9764.CRC-25-0563)
Supplement: Supplementary Figure S9 — Figure S9. Ingenuity Pathway analysis indicated that the activities of several upstream regulators important for meningioma growth were significantly altered in AG-NF2-Men cells treated with the brigatinib+INK128 combination. [file crc-25-0563_supplementary_figure_s9_suppsf9.pdf]

Supplementary Figure S9. Ingenuity Pathway analysis indicated that the activities of several upstream regulators important for meningioma growth were significantly altered in AG-NF2-Men cells treated with the brigatinib+INK128 combination. Displayed are selected upstream regulators and the log2 fold-change of their transcripts (relative to the DMSO control) expressed in the brigatinib+INK128 combination-treated AG-NF2-Men cells. The activation z-score indicates the predicted activation state of a given upstream regulator, with z-scores ≥ 2 indicating predicted activation and z-scores ≤ -2 indicating predicted inhibition. The P-value of overlap was calculated with Fisher's Exact Test and represents the statistical significance of overlap between gene expression changes in the combination-treated cells versus known target genes of the listed upstream regulator, signifying the likelihood that the observed transcriptional changes were affected by the upstream regulator. The list is grouped by the proposed biological function of the regulators in meningioma cells. The full list of statistically significant activated and inhibited upstream regulators in the brigatinib+INK128 combination-treated AG-NF2-Men cells is shown in Supplementary Data S2, as well as lists for single-agent brigatinib and INK128 treatments.

Upstream regulator	Log2 fold-change	Activation z-score	Predicted activation state	P-value of overlap	Biological function
NRG1	-2.93	-2.447	Inhibited	5.99E-04	EGFR/ErbB signaling
EGFR	-1.26	-3.211	Inhibited	5.31E-07	EGFR/ErbB signaling
ERBB2	-0.88	-5.691	Inhibited	6.97E-40	EGFR/ErbB signaling
ERBB3	-2.4	-3.769	Inhibited	9.24E-09	EGFR/ErbB signaling
AKT (family)		-2.378	Inhibited	2.32E-05	Downstream target of RTKs and non-RTKs
PAK1	-1.13	-2.092	Inhibited	8.43E-03	Downstream target of RTKs and non-RTKs
YAP/TAZ (family)		-2.247	Inhibited	1.52E-02	HIPPO/YAP/TAZ/TEAD signaling
LATS2	-0.43	2.764	Activated	5.29E-02	HIPPO/YAP/TAZ/TEAD signaling
LATS1	-0.28	2.138	Activated	1.02E-01	HIPPO/YAP/TAZ/TEAD signaling
YAP1	-0.16	-4.048	Inhibited	4.28E-19	HIPPO/YAP/TAZ/TEAD signaling
TEAD1	-0.38	-2.88	Inhibited	3.47E-11	HIPPO/YAP/TAZ/TEAD signaling
TEAD (family)		-2.987	Inhibited	3.87E-04	HIPPO/YAP/TAZ/TEAD signaling
FOXM1	-0.77	-4.467	Inhibited	4.81E-04	Overexpression correlates with aggressive meningioma behavior
ZEB1	-0.61	-2.233	Inhibited	5.19E-04	Transcription factor involved in EMT
ZEB2	0.3	-2.202	Inhibited	7.35E-04	Transcription factor involved in EMT
MYC	-2.39	-3.809	Inhibited	3.97E-18	Regulate cell growth, apoptosis, differentiation, and metabolism
FOSL1	-6.15	-3.049	Inhibited	1.48E-04	Implicated as a regulator of cell proliferation and differentiation
YY1	-0.06	-2.114	Inhibited	2.40E-01	Regulate gene transcription involved in cell proliferation and differentiation
TP53	-0.65	5.65	Activated	4.91E-20	Regulate cell cycle progression
CDKN1A	1.83	3.152	Activated	7.88E-09	Cyclin-dependent kinase inhibitor
MUC1	0.17	-2.663	Inhibited	6.24E-05	Cytoplasmic domain represses p53 signaling
