# Supplementary material for: Combining Brigatinib with mTOR Inhibition to Effectively Treat NF2-SWN–Associated and Sporadic NF2-Deficient Meningiomas
Source: Cancer Res Commun. 2026 Jan 27;6(1):211–23. doi: 10.1158/2767-9764.CRC-25-0563 (PMC12835584; doi:10.1158/2767-9764.CRC-25-0563)

Supplementary Figure S10. AG-NF2-Men-Luc2 cells did not grow when implanted subcutaneously but readily established intracranial xenografts when injected into the skull base of NSG mice. (A) AG-NF2-Men-Luc2 cells were injected subcutaneously in the back of NSG mice, followed by BLI to monitor tumor growth. The tumor-emitted luminescence was captured every two weeks and the average relative luciferase units (RLU) of tumor-emitted BL signal for the entire group (n=5) was calculated and denoted as % of total flux relative to that of the first scan at two weeks after cell injection designated as one (100%). (B) AG-NF2-Men-Luc2 cells were stereotactically injected into the skull base of NSG mice and tumor-emitted luminescence captured every week. The mean RLU of tumor-emitted BL signal for the entire group was calculated relative to the first scan at one week after engraftment (designated as one). Graphs are plotted as mean ± SE.

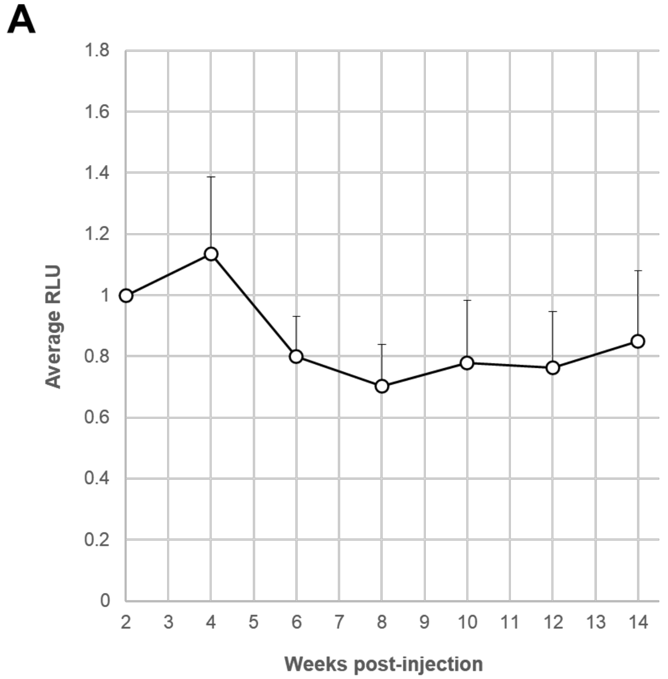

Supplement: Supplementary Figure S10 — Figure S10. AG-NF2-Men-Luc2 cells did not grow when implanted subcutaneously but readily established intracranial xenografts when injected into the skull base of NSG mice. [file crc-25-0563_supplementary_figure_s10_suppsf10.pdf]
