# Supplementary material for: Combining Brigatinib with mTOR Inhibition to Effectively Treat NF2-SWN–Associated and Sporadic NF2-Deficient Meningiomas
Source: Cancer Res Commun. 2026 Jan 27;6(1):211–23. doi: 10.1158/2767-9764.CRC-25-0563 (PMC12835584; doi:10.1158/2767-9764.CRC-25-0563)
Supplement: Supplementary Figure S11 — Figure S11. The brigatinib+INK128 combination more effectively shrank intracranial AG. [file crc-25-0563_supplementary_figure_s11_suppsf11.pdf]

Supplementary Figure S11. The brigatinib+INK128 combination more effectively shrank intracranial AG-NF2-Men-Luc2 meningioma xenografts than brigatinib or INK128 alone. (Top) The graph shows the relative tumor-emitted luminescence signals, denoted as % of total flux after treatment relative to the total flux prior to treatment designated as one (100%), in mice treated with brigatinib, INK128, or their combination. Data are depicted as means with SEMs for each timepoint. (Bottom) Statistical pairwise comparisons of the AG-NF2-Men-Luc2 xenografts treated with vehicle, brigatinib, INK128, or brigatinib+INK128 shown in Figure 6 and Table 1. P-values were calculated using the cross-sectional analysis tab of the TumGrowth website (https://kroemerlab.shinyapps.io/TumGrowth/) after log transformation of normalized tumor luminescence.

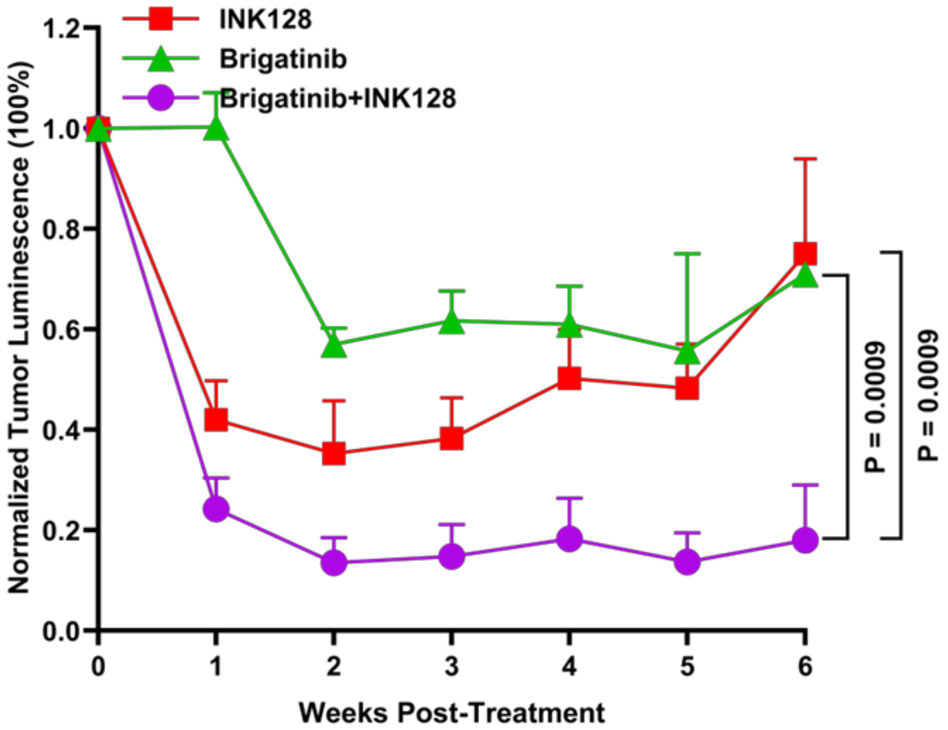

Pairwise comparisons of AG-NF2-Men-Luc2 xenografts

Treatment 1	Treatment 2	P-value
Brigatinib	Vehicle	<0.0001
INK128	Vehicle	<0.0001
Brigatinib+INK128	Vehicle	<0.0001
INK128	Brigatinib	0.9970
Brigatinib+INK128	Brigatinib	0.0009
Brigatinib+INK128	INK128	0.0009
