# Supplementary material for: Combining Brigatinib with mTOR Inhibition to Effectively Treat NF2-SWN–Associated and Sporadic NF2-Deficient Meningiomas
Source: Cancer Res Commun. 2026 Jan 27;6(1):211–23. doi: 10.1158/2767-9764.CRC-25-0563 (PMC12835584; doi:10.1158/2767-9764.CRC-25-0563)

Supplementary Figure S12. Combining INK128 with brigatinib also enhanced anti-tumor effects in the sporadic *NF2*-deficient Ben-Men-1-LucB xenograft model. Mice with established intracranial Ben-Men-1-LucB xenografts were treated with vehicle, brigatinib, INK128, or brigatinib+INK128 by oral gavage, and tumor growth was monitored by weekly BLI as described in Supplementary Methods. The relative tumor-emitted BL signals were quantified and denoted as % of total flux after treatment relative to the total flux prior to treatment designated as one (100%). The data are shown as mean ± SE.

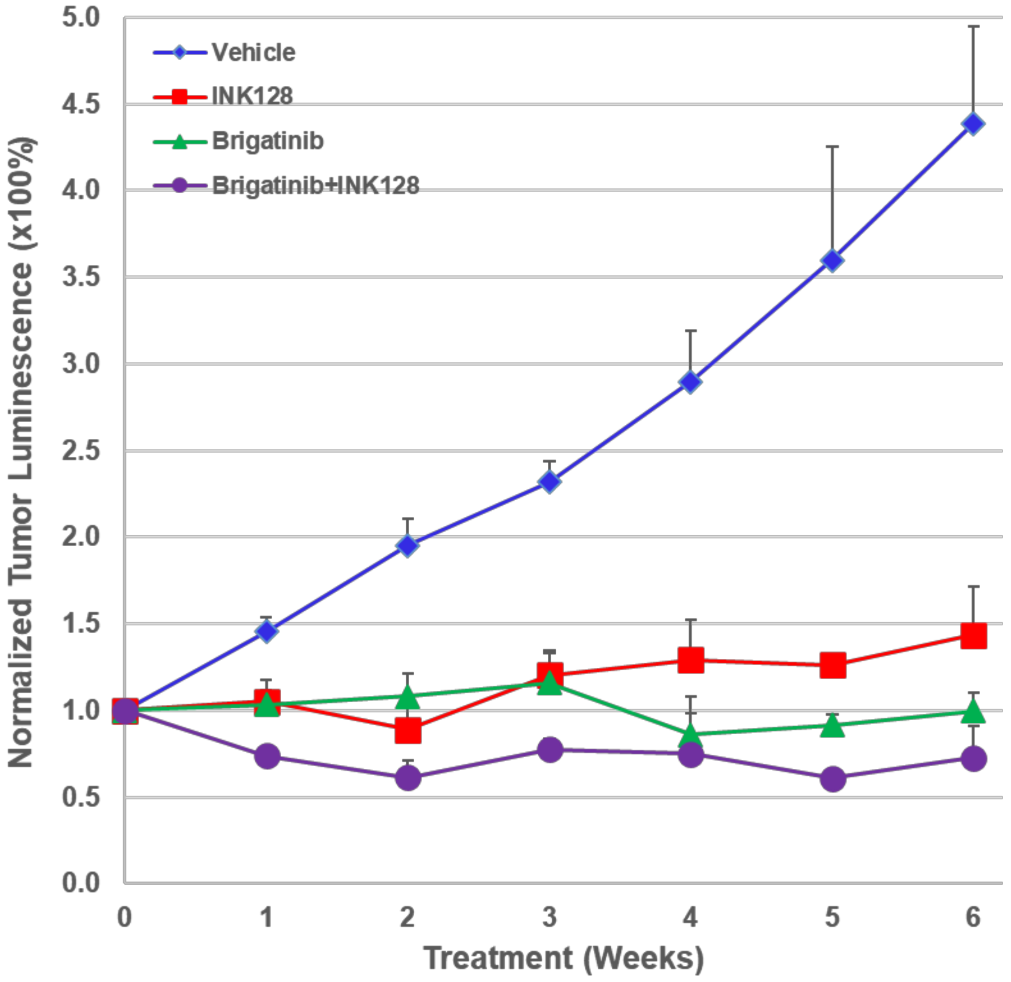

Supplement: Supplementary Figure S12 — Figure S12. Combining INK128 with brigatinib also enhanced anti-tumor effects in the sporadic NF2-deficient Ben-Men-1-LucB xenograft model. [file crc-25-0563_supplementary_figure_s12_suppfs12.pdf]
