# Supplementary material for: Combining Brigatinib with mTOR Inhibition to Effectively Treat NF2-SWN–Associated and Sporadic NF2-Deficient Meningiomas
Source: Cancer Res Commun. 2026 Jan 27;6(1):211–23. doi: 10.1158/2767-9764.CRC-25-0563 (PMC12835584; doi:10.1158/2767-9764.CRC-25-0563)

Supplementary Figure S13. Treatment with brigatinib, INK128, or brigatinib+INK128 did not cause overt weight loss, compared with vehicle-treated mice. Meningioma xenograft-bearing mice were treated with vehicle, brigatinib, INK128, or brigatinib+INK128 by oral gavage every day for eight weeks. Mouse weights were measured every day. Shown are the mean + SEM for each group of mice.

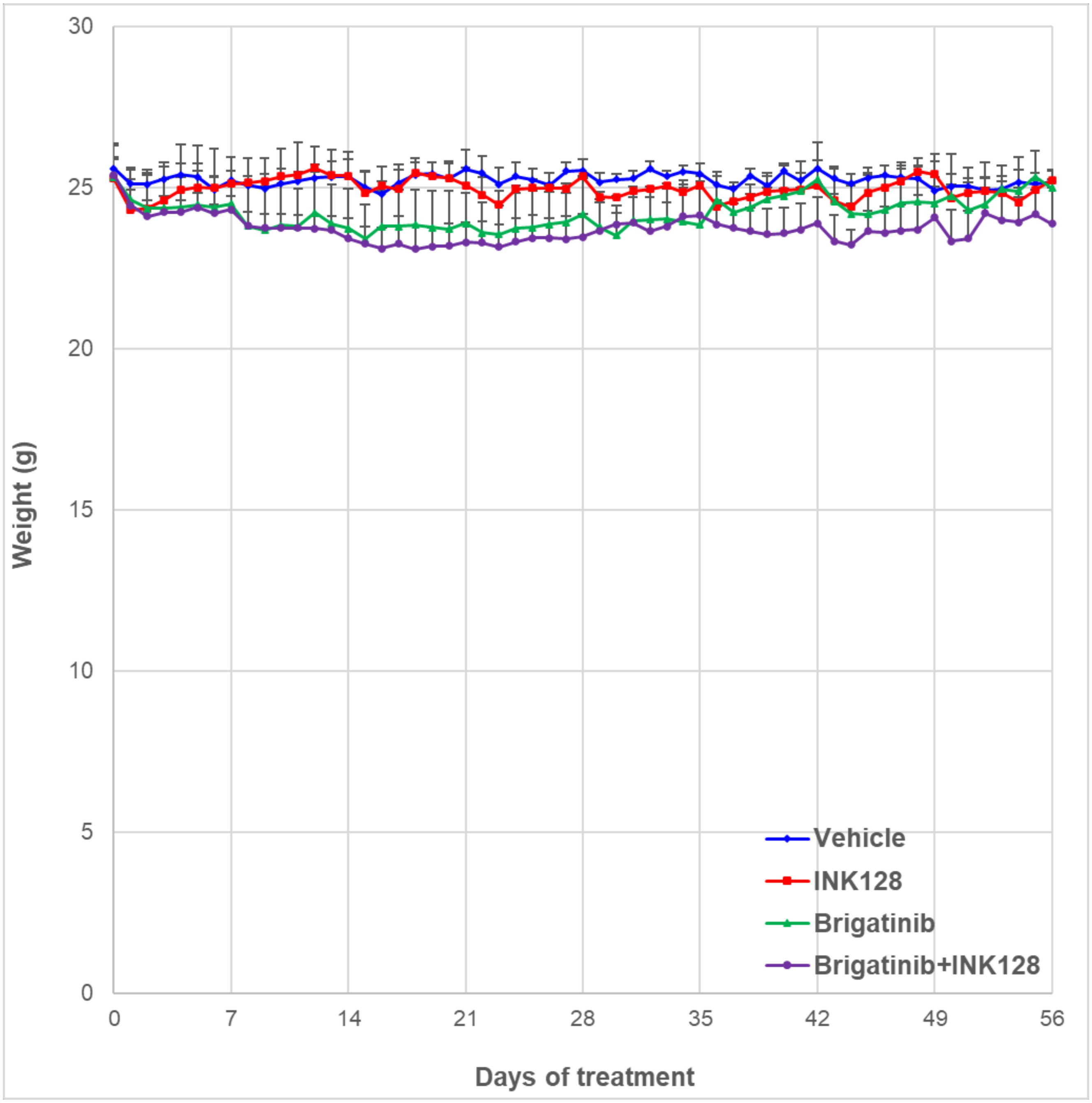

Supplement: Supplementary Figure S13 — Figure S13. Treatment with brigatinib, INK128, or brigatinib+INK128 did not cause overt weight loss, compared with vehicle-treated mice. [file crc-25-0563_supplementary_figure_s13_suppsf13.pdf]
